# Use of machine learning to analyse routinely collected intensive care unit data: a systematic review

**DOI:** 10.1186/s13054-019-2564-9

**Published:** 2019-08-22

**Authors:** Duncan Shillan, Jonathan A. C. Sterne, Alan Champneys, Ben Gibbison

**Affiliations:** 1 NIHR Bristol Biomedical Research Centre, University of Bristol, Bristol, UK; 2 Population Health Sciences, Bristol Medical School, University of Bristol, Bristol, UK; 3 Department of Engineering Mathematics, University of Bristol, Bristol, UK; 4 Translational Health Sciences, Bristol Medical School, University of Bristol, Bristol, UK; 5 Department of Anaesthesia, Bristol Royal Infirmary, Level 7 Queens Building, Upper Maudlin St, Bristol, BS2 8HW UK

**Keywords:** Artificial intelligence, Machine learning, Intensive care unit, Routinely collected data

## Abstract

**Background:**

Intensive care units (ICUs) face financial, bed management, and staffing constraints. Detailed data covering all aspects of patients’ journeys into and through intensive care are now collected and stored in electronic health records: machine learning has been used to analyse such data in order to provide decision support to clinicians.

**Methods:**

Systematic review of the applications of machine learning to routinely collected ICU data. Web of Science and MEDLINE databases were searched to identify candidate articles: those on image processing were excluded. The study aim, the type of machine learning used, the size of dataset analysed, whether and how the model was validated, and measures of predictive accuracy were extracted.

**Results:**

Of 2450 papers identified, 258 fulfilled eligibility criteria. The most common study aims were predicting complications (77 papers [29.8% of studies]), predicting mortality (70 [27.1%]), improving prognostic models (43 [16.7%]), and classifying sub-populations (29 [11.2%]). Median sample size was 488 (IQR 108–4099): 41 studies analysed data on > 10,000 patients. Analyses focused on 169 (65.5%) papers that used machine learning to predict complications, mortality, length of stay, or improvement of health. Predictions were validated in 161 (95.2%) of these studies: the area under the ROC curve (AUC) was reported by 97 (60.2%) but only 10 (6.2%) validated predictions using independent data. The median AUC was 0.83 in studies of 1000–10,000 patients, rising to 0.94 in studies of > 100,000 patients. The most common machine learning methods were neural networks (72 studies [42.6%]), support vector machines (40 [23.7%]), and classification/decision trees (34 [20.1%]). Since 2015 (125 studies [48.4%]), the most common methods were support vector machines (37 studies [29.6%]) and random forests (29 [23.2%]).

**Conclusions:**

The rate of publication of studies using machine learning to analyse routinely collected ICU data is increasing rapidly. The sample sizes used in many published studies are too small to exploit the potential of these methods. Methodological and reporting guidelines are needed, particularly with regard to the choice of method and validation of predictions, to increase confidence in reported findings and aid in translating findings towards routine use in clinical practice.

**Electronic supplementary material:**

The online version of this article (10.1186/s13054-019-2564-9) contains supplementary material, which is available to authorized users.

## Key messages

Publication of papers reporting the use of machine learning to analyse routinely collected ICU data is increasing rapidly: around half of the identified studies were published since 2015.

Machine learning methods have changed over time. Neural networks are being replaced by support vector machines and random forests.

The majority of published studies analysed data on fewer than 1000 patients. Predictive accuracy increased with increasing sample size.

Reporting of the validation of predictions was variable and incomplete—few studies validated predictions using independent data.

Methodological and reporting guidelines may increase confidence in reported findings and thereby facilitate the translation of study findings towards routine use in clinical practice.

## Background

Intensive care units (ICUs) face financial, bed management, and staffing constraints among others. Efficient operation in the light of these limits is difficult because of their multidimensional and interconnected nature [1]. Extremely detailed data covering all aspects of patients’ journeys into and through intensive care are now collected and stored in electronic health records (EHRs). Data that are typically available in these EHRs include demographic information, repeated physiological measurements, clinical observations, laboratory test results, and therapeutic interventions. Such detailed data offer the potential to provide improved prediction of outcomes such as mortality, length of stay, and complications, and hence improve both the care of patients and the management of ICU resources [2–4].

Machine learning is a form of artificial intelligence (AI) in which a model learns from examples rather than pre-programmed rules. Example inputs and output for a task are provided to ‘the machine’ and, using learning algorithms, a model is created so that new information can be interpreted. Machine learning approaches can provide accurate predictions based on large, structured datasets extracted from EHRs [5, 6]. There have been rapid developments in machine learning methodology, but many methods still require large datasets to model complex and non-linear effects, and thereby improve on prediction rules developed using standard statistical methods [6–8]. Papers describing applications of machine learning to routinely collected data are published regularly [7], but there is no recent systematic review summarizing their characteristics and findings [9]. We systematically reviewed the literature on uses of machine learning to analyse routinely collected ICU data with a focus on the purposes of the application, type of machine learning methodology used, size of the dataset, and accuracy of predictions.

## Methods

### Systematic review design, definitions, and inclusion/exclusion criteria

#### Search strategy

Candidate articles were identified from searches of Web of Science and MEDLINE. There was no restriction on the publication date, but only articles written in English were included. Two searches connected with an ‘AND’ statement were used—one to capture applications of artificial intelligence and the other to capture the ICU setting. Searches for artificial intelligence used the following terms: ‘Machine Learning’, ‘Artificial Intelligence’, ‘Deep Learning’, ‘Neural Network’, ‘Support vector machine’, ‘Prediction Network’, ‘Forecast Model’, ‘Data mining’, ‘Supervised Learning’, and ‘Time series prediction’. Searches for the applications of artificial intelligence use the following terms: ‘Cardiac Intensive Care Unit’, ‘CICU’, ‘ICU’, ‘Coronary Care’, ‘Critical Care’, ‘High Dependency’, and ‘HDU’. The search terms were made in an iterative process, initially using subject headings from citation indexes and text word searches for machine learning (e.g. ‘Artificial Intelligence/or Machine Learning/’, ‘Pattern Recognition, Automated/’, and ‘Machine learning.tw’, ‘Artificial intelligence.tw’, ‘Deep learning.tw’, ‘Supervised learning.tw’ respectively). The first 30 relevant papers were extracted and mined for specific terms (e.g. ‘Prediction network.tw’, ‘Support vector machine?.tw’, ‘Demand Forecasting.tw.’). The search was run again with these terms included, and the first 30 new relevant papers were extracted and mined for specific terms. These were included in the search terms to generate the final list of search terms (see Additional file 1). Review papers were set aside for separate analysis.

#### Eligibility criteria

Eligible papers (1) used machine learning or artificial intelligence (AI), defined as any form of automated statistical analysis or data science methodology; (2) analysed routinely collected data that were generated as part of patients’ standard care pathway in any hospital worldwide; (3) analysed data collected in the ICU, defined as an area with a sole function to provide advanced monitoring or support to single or multiple body systems; and (4) were published in a scientific journal or conference proceeding when the proceeding detailed the full study. Studies from all ICUs were eligible, regardless of specialty. There was no limit on the age of included patients. The following types of study were excluded: (1) use of machine learning to process or understand medical images; (2) studies that focused on text mining; (3) analyses of novel research data rather than routinely collected data; (4) studies that implemented additional data collection techniques beyond hospitals’ routine systems; (5) studies based on data from a general medicine ward, coronary care unit, operating theatre or post-anaesthetic care unit, or emergency room; (6) conference abstracts and proprietary machine learning systems. Papers describing reviews of machine learning based on ICU data were also retrieved.

#### Study selection

Details of papers were uploaded to EndNote X8 (Clarivate Analytics, Philadelphia, PA, USA), and duplicates were removed using EndNote’s duplicate identification tool. One author (DS) screened the titles and abstracts and retrieved the full text of papers judged to be potentially eligible for the review. Final decisions about eligibility, based on reading the full text of the manuscripts, were made by one author (DS), with a randomly selected subset checked by two further authors (BG and JS). Conflicts were resolved by consensus. An additional file provides a full bibliography (see Additional file 2).

#### Review process and data extraction

The study characteristics to be extracted, and their definitions and categories, were decided iteratively following study of 40 eligible papers. We extracted information on the following study features: (1) aim (categorized as improving prognostic models, classifying sub-populations, determining physiological thresholds of illness, predicting mortality, predicting length of stay, predicting complications, predicting health improvement, detecting spurious values, alarm reduction, improving upon previous methods (with details) and other (with details)); (2) type of machine learning (categorized as classification/decision trees, naïve Bayes/Bayesian networks, fuzzy logic, Gaussian process, support vector machine, random forest, neural network, superlearner, not stated and other (with details)). All types of machine learning used were recorded; (3) dataset size (the number of patients, episodes or samples analysed); (4) whether the study used data from the publicly available Medical Information Mart for Intensive Care II/III (MIMIC-II/III), which includes deidentified health data on around 40,000 patients treated at the Beth Israel Deaconess Medical Center between 2001 and 2012 [10]; (5) method used to validate predictions (categorized as independent data, randomly selected subset with leave-P-out (P recorded), *k*-fold cross-validation (*k* recorded), randomly selected subset, other (with details), no validation). For studies that validated results for multiple machine learning techniques, we recorded the method corresponding to the most accurate approach. For studies that dichotomized length of stay in order to validate predictions, we recorded the threshold as the highest length of stay in the lower-stay group; (6) measure of predictive accuracy (area under the receiver operator characteristic (ROC) curve (AUC): proportion of subjects correctly classified, sensitivity, and specificity). Each measure reported was recorded. When measures of predictive accuracy were recorded for multiple machine learning techniques, we recorded the measures for the most accurate approach. For multiple outcomes, we recorded the measures corresponding to the longest-term outcome. When multiple validation datasets were used, we recorded the measures for the most accurate approach; (7) reporting of results from standard statistical methods such as linear regression and logistic regression. We recorded the method and the corresponding measures of accuracy, using the same rules as described above when more than one result was reported. In response to a suggestion from a peer reviewer, we recorded whether papers reported on calibration and, if so, the method that was used.

Risk of bias was not assessed in the included studies because the purpose of our review was descriptive—the aim was not to draw conclusions about the validity of estimates of predictive accuracy from the different studies. The size of dataset analysed was tabulated according to the study aims. Analyses were restricted to studies that used machine learning to predict complications, mortality, length of stay, or health improvement. The size of dataset according to the type of machine learning, the approach to validation according to outcome predicted, and the measure of predictive accuracy according to outcome predicted were tabulated. The distribution of AUC according to the number of patients analysed and outcome predicted was plotted, along with the number of papers published according to the type of machine learning and year of publication.

## Results

### Identification of eligible studies

**Fig. 1:**
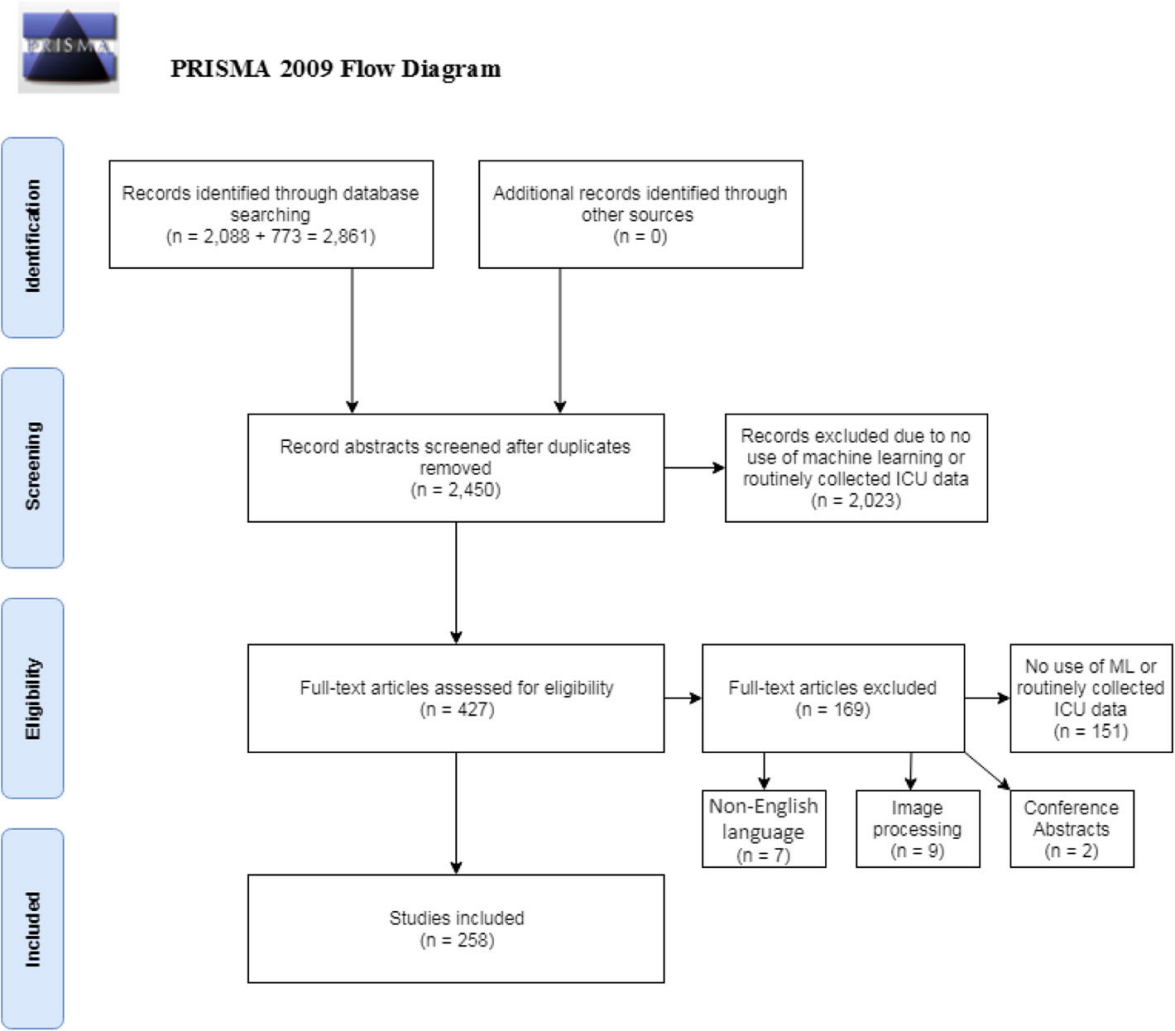
PRISMA 2009 flow diagram of study review process and exclusion of papers. From [11]

Two thousand eighty-eight papers were identified through Web of Science and 773 through MEDLINE. After duplicates were removed, the titles and abstracts of 2450 unique papers were screened, of which 2023 papers were classified as ineligible. Of 427 papers for which the full text was reviewed, 169 were found to be ineligible, mainly because they did not use machine learning or did not analyse routinely collected ICU data. The review therefore included 258 papers (Fig. 1). MIMIC-II/III data were used in 63 (24.4%) of these studies.

### Purpose of machine learning in the ICU

The most common study aims were predicting complications (77 papers [29.8% of studies]), predicting mortality (70 [27.1%]), improving prognostic models (43 [16.7%]), and classifying sub-populations (29 [11.2%]) (Table 1). The median sample size across all studies was 488 (IQR 108–4099). Only six studies (three predicting complications, two improving prognostic models, and one predicting mortality) analysed data on more than 100,000 patients, while 35 analysed data on 10,000–100,000 patients, 18 (51.4%) of which attempted to predict mortality. Most studies (211 [81.8%]) reported analyses of fewer than 10,000 patients. Large sample sizes (> 10,000 patients) were most frequent in studies predicting complications, mortality, or length of stay, and those aiming to improve prognostic models or risk scoring systems. Sample sizes were usually less than 1000 in studies determining physiological thresholds or that aimed to detect spurious values.

**Table 1:** Number and proportion of papers according to the aim of study and number of patients analysed

		Number of patients analysed					
Aim of study	Number (%) of papers with this aim^a^	< 100	100–1000	1000–10,000	10,000–100,000	100,000–1,000,000	Number not reported
Predicting complications	79 (30.6%)	23 (29.1%)	26 (32.9%)	17 (21.5%)	8 (10.1%)	3 (3.8%)	2 (2.5%)
Predicting mortality	70 (27.1%)	11 (15.7%)	19 (27.1%)	19 (27.1%)	18 (25.7%)	1 (1.4%)	2 (2.9%)
Improving prognostic models/risk scoring system	43 (16.7%)	8 (18.6%)	16 (37.2%)	8 (18.6%)	8 (18.6%)	2 (4.7%)	1 (2.3%)
Classifying sub-populations	29 (11.2%)	11 (37.9%)	8 (27.6%)	6 (20.7%)	2 (6.9%)	0 (0.0%)	2 (6.9%)
Alarm reduction	21 (8.14%)	9 (42.9%)	5 (23.8%)	7 (33.3%)	0 (0.0%)	0 (0.0%)	0 (0.0%)
Predicting length of stay	18 (6.98%)	3 (16.7%)	7 (38.9%)	5 (27.8%)	3 (16.7%)	0 (0.0%)	0 (0.0%)
Predicting health improvement	17 (6.59%)	5 (29.4%)	10 (58.8%)	2 (11.8%)	0 (0.0%)	0 (0.0%)	0 (0.0%)
Determining physiological thresholds	16 (6.20%)	10 (62.5%)	4 (25.0%)	1 (6.2%)	0 (0.0%)	0 (0.0%)	1 (6.2%)
Improving upon previous methods	5 (1.94%)	2 (40.0%)	1 (20.0%)	1 (20.0%)	1 (20.0%)	0 (0.0%)	0 (0.0%)
Detecting spurious recorded values	3 (1.16%)	1 (33.3%)	2 (66.7%)	0 (0.0%)	0 (0.0%)	0 (0.0%)	0 (0.0%)
Total (accounting for duplicates)	258	72 (27.9%)	84 (32.6%)	55 (21.3%)	35 (13.6%)	6 (2.33%)	6 (2.33%)

^a^Where papers had more than one aim, all aims were recorded, so percentages may total more than 100

All further analyses were restricted to the 169 studies that predicted at least one of four clearly definable types of outcome: complications, mortality, length of stay, and health improvement. MIMIC-II/III data were used in 45 (26.6%) of these 169 studies, a similar rate to the use of MIMIC-II/III data in all outcomes (63 [24.4%]).

### Type of machine learning

Among studies that predicted complications, mortality, length of stay, or health improvement, 12 (7.1%) predicted more than one of these types of outcome (Table 2). The most commonly used types of machine learning were neural networks (72 studies [42.6%]), support vector machines (40 [23.7%]), and classification/decision trees (34 [20.1%]). The median sample size was 863 (IQR 150–5628). More than half of the studies analysed data on fewer than 1000 patients. There were no strong associations between the type of machine learning and size of dataset, although the proportion of studies with sample sizes less than 1000 was the highest for those using support vector machines and fuzzy logic/rough sets. Machine learning methods used in fewer than five papers were combined under the “Other” category: of these. Data on the machine learning methods used in the different types of prediction study are available from the authors on request.

**Table 2:** Number and proportion of papers according to the type of machine learning used and number of patients analysed (for prediction studies only)

		Number of patients analysed					
Type of machine learning	Number (%) of papers with this type^a^	< 100	100–1000	1000–10,000	10,000–100,000	100,000–1,000,000	Number not reported
Neural network	72 (42.6%)	14 (19.4%)	27 (37.5%)	20 (27.8%)	9 (12.5%)	2 (2.8%)	0 (0.0%)
Support vector machine	40 (23.7%)	12 (30.0%)	15 (37.5%)	8 (20.0%)	4 (10.0%)	1 (2.5%)	0 (0.0%)
Classification/decision trees	35 (20.7%)	6 (17.1%)	11 (31.4%)	10 (28.6%)	5 (14.3%)	1 (2.9%)	2 (5.7%)
Random forest	21 (12.4%)	1 (4.8%)	9 (42.9%)	5 (23.8%)	4 (19.0%)	2 (9.5%)	0 (0.0%)
Naive Bayes/Bayesian networks	19 (11.2%)	4 (21.1%)	5 (26.3%)	6 (31.6%)	2 (10.5%)	1 (5.3%)	1 (5.3%)
Fuzzy logic/rough set	12 (7.1%)	3 (25.0%)	5 (41.7%)	2 (16.7%)	1 (8.3%)	0 (0.0%)	1 (8.3%)
Other techniques^b^	28 (16.7%)	2 (7.1%)	10 (35.7%)	8 (28.6%)	7 (25.0%)	1 (3.6%)	0 (0.0%)
Total (accounting for duplicates)	169	37 (21.9%)	56 (33.1%)	42 (24.9%)	26 (15.4%)	4 (2.37%)	4 (2.37%)

^a^Papers can have more than one approach—percentages may total more than 100

^b^Other techniques (number of studies): causal phenotype discovery (1), elastic net (1), factor analysis (1), Gaussian process (2), genetic algorithm (1), hidden Markov models (1), InSight (4); JITL-ELM (1), k-nearest neighbour (3), Markov decision process (1), particle swarm optimization (1), PhysiScore (1), radial domain folding (1), sequential contrast patterns (1), Superlearner (4), switching linear dynamical system (1), Weibull-Cox proportional hazards model (1), method not described (2)

**Fig. 2:**
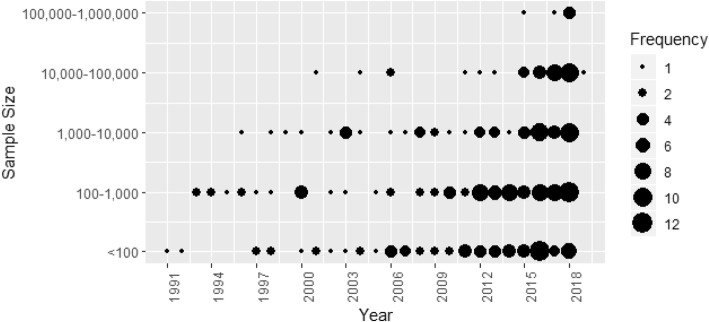
Number of papers published according to the sample size and year of publication

Machine learning studies using ICU data were published from 1991 onwards (Fig. 2). The earliest studies were based on fewer than 100 patients: the first studies based on more than 1000, 10,000, and 100,000 patients were published in 1996, 2001, and 2015 respectively. Although study sizes have increased over time (among studies published in 2017 and 2018, the median [IQR] sample size was 3464 [286–21,498]), studies based on fewer than 1000 patients have been regularly published in recent years. Six studies used data on more than 100,000 patients: one in 2015, one in 2017, and four in 2018 [2, 12–16].

**Fig. 3:**
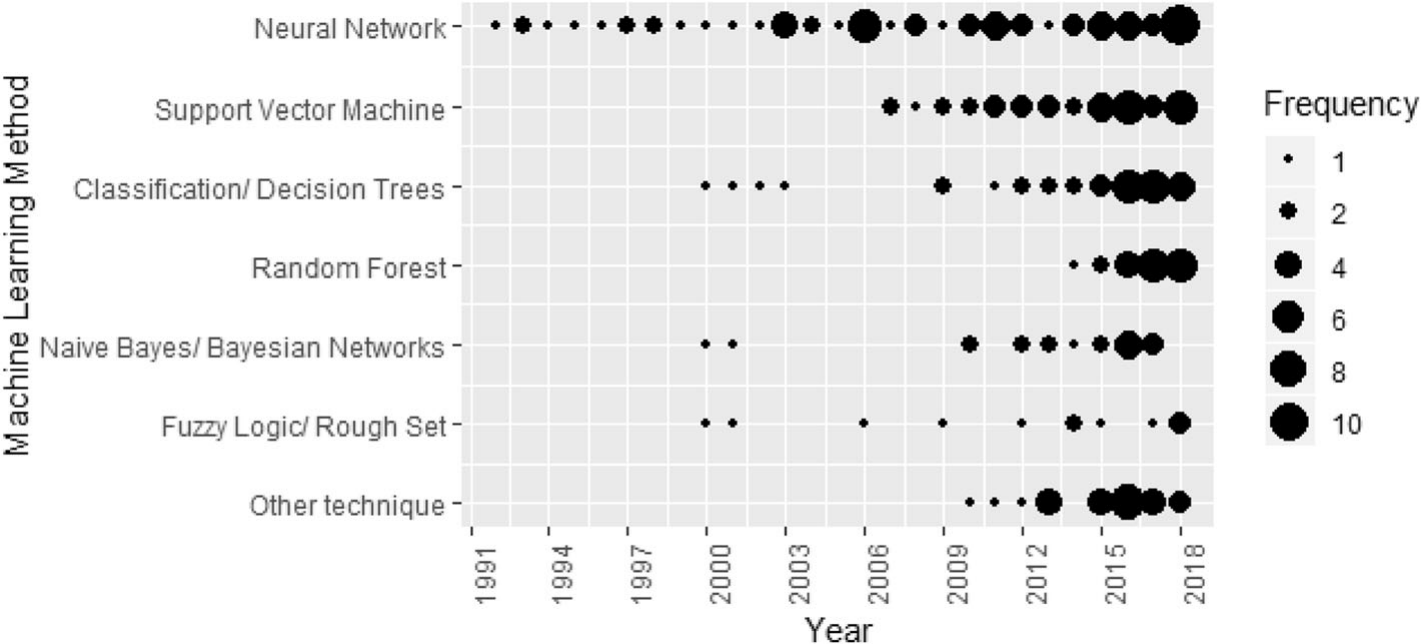
Number of papers published according to the type of machine learning and year of publication

The earliest machine learning studies all used neural networks (Fig. 3). Papers using other machine learning methods were reported from 2000 onwards, with support vector machines reported from 2005 and random forests from 2012. Of the 258 studies, 125 (48%) were published from 2015 onwards. The most commonly reported machine learning approaches in these studies were support vector machines (37 [29.6% of recent studies]), random forests (29 [23.2%]), neural networks (31[24.8%]), and classification/decision trees (27 [21.6%]).

### Approaches to validation

Table 3 shows that of 169 studies that predicted complications, mortality, length of stay, or health improvement, 161 (95.2%) validated the predictions. Validations were rarely based on independent data (10 studies [6.2%]). The most commonly used approaches were to use random subsets of the data with (71 [44.1%]) or without (71 [44.1%]) *k*-fold cross-validation respectively. Studies predicting the length of stay were most likely to use independent data and least likely to use *k*-fold cross-validation. Data on approach to validation according to the type of machine learning and outcome predicted are available from the authors on request.

**Table 3:** Number and proportion of papers according to outcome predicted and approach to validation (for prediction studies only)

			Approach to validation^b^				
Outcome predicted	Total papers^a^	Validated	Independent data	Leave-P-out	*k*-fold cross-validation	Randomly selected subset	Other^b^
Complications	79 (46.7%)	73 (92.4%)	5 (6.85%)	5 (6.85%)	33 (45.2%)	30 (41.1%)	0 (0%)
Mortality	70 (41.4%)	68 (97.1%)	5 (7.35%)	3 (4.41%)	33 (48.5%)	27 (39.7%)	0 (0%)
Length of stay	18 (10.7%)	18 (100%)	3 (16.7%)	1 (5.56%)	4 (22.2%)	10 (55.6%)	1 (5.6%)
Health improvement	17 (10.1%)	16 (94.1%)	0 (0%)	1 (6.25%)	5 (31.2%)	10 (56.2%)	0 (0%)
Total (accounting for duplicates)	169	161 (94.1%)	10 (6.2%)	8 (5%)	71 (44.1%)	71 (44.1%)	1 (0.6%)

^a^Papers can have more than one approach, so percentages may total more than 100

^b^“Other” techniques (number of studies): a comparison between ML and decisions made by clinicians (1)

### Measures of predictive accuracy reported

The majority of the 161 papers that quantified the predictive accuracy of their algorithm reported the AUC (97 [60.2%]), of which 43 (26.7%) papers also reported accuracy, sensitivity, and specificity (Table 4). Sixty-two studies (38.5%) reported these measures but not the AUC. The AUC was most likely to be reported by studies predicting mortality (47 [69.1%]). Papers predicting complications and health improvement were more likely to report only accuracy, sensitivity, and specificity. All 18 papers predicting the numerical outcome of length of stay validated their predictions: 8 (44.4%) reported the proportion of variance explained (*R*^2^). There were 5 papers that reported the AUC dichotomized length of stay: two papers at 1 day [17, 18], one at 2 days [19], one at 7 days [4], and one at 10 days [20]. Data on reported measures of predictive accuracy according to the type of machine learning and outcome predicted are available from the authors on request.

**Table 4:** Number and proportion of papers according to outcome predicted and measure of predictive accuracy reported (for studies that validated predictions)

		Measure of predictive accuracy reported^a^				
Outcome predicted	Total papers	AUC and accuracy/sensitivity/specificity	AUC only	Accuracy/sensitivity/specificity only	*R* ^2^	Other^b^
Complication	73 (45.3%)	24 (32.9%)	17 (23.3%)	28 (38.4%)		4 (5.5%)
Mortality	68 (42.2%)	16 (23.5%)	31 (45.6%)	18 (26.5%)		3 (4.4%)
Length of stay	18 (11.1%)	2 (11.8%)	3 (16.7%)	5 (27.8%)	8 (44.4%)	1 (5.6%)
Health improvement	16 (10%)	1 (6.3%)	3 (18.8%)	11 (68.8%)		1 (6.3%)
Total	161	43 (26.7%)	54 (33.5%)	62 (38.5%)	8 (5.0%)	9 (5.6%)

^a^Papers can have more than one approach, so percentages may total more than 100. The total of these columns does not account for duplicates as papers can fluctuate how they discuss different results

^b^“Other” measures of predictive accuracy (number): congruence of ML and clinician’s decisions (1), Matthews correlation coefficient (1), mean absolute differences between observed and predicted (1), mean error rate (1), MSE as loss function (1), Pearson correlation between estimate and actual (1), ratio of wins vs loses against logistic regression (1), rules developed by ML (1)

**Fig. 4:**
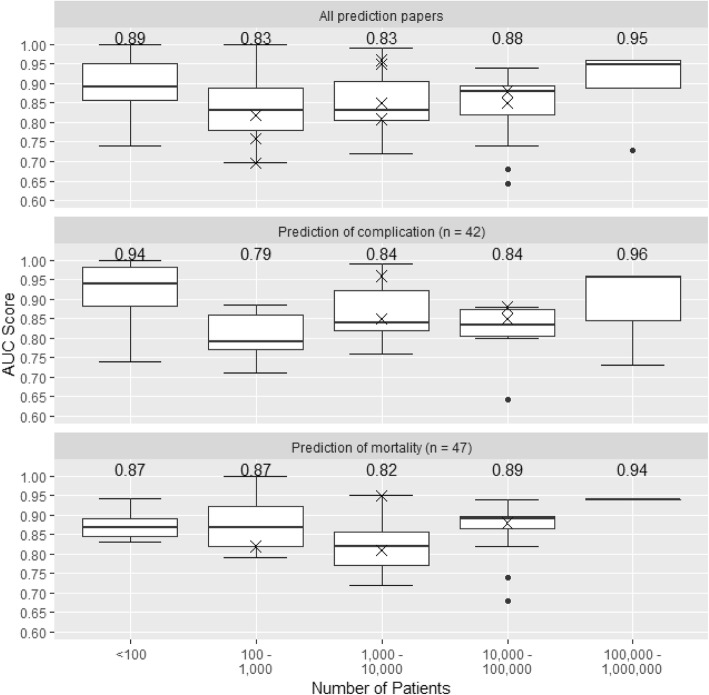
Boxplots showing the distribution of AUC scores according to the size of dataset, for all studies and separately for studies predicting mortality and complications. Numbers displayed are the median AUC for each group. A cross indicates the AUC of one of the 10 papers using independent test data. We did not plot results for studies predicting the length of stay and health improvement because the numbers of such studies were small

**Fig. 5:**
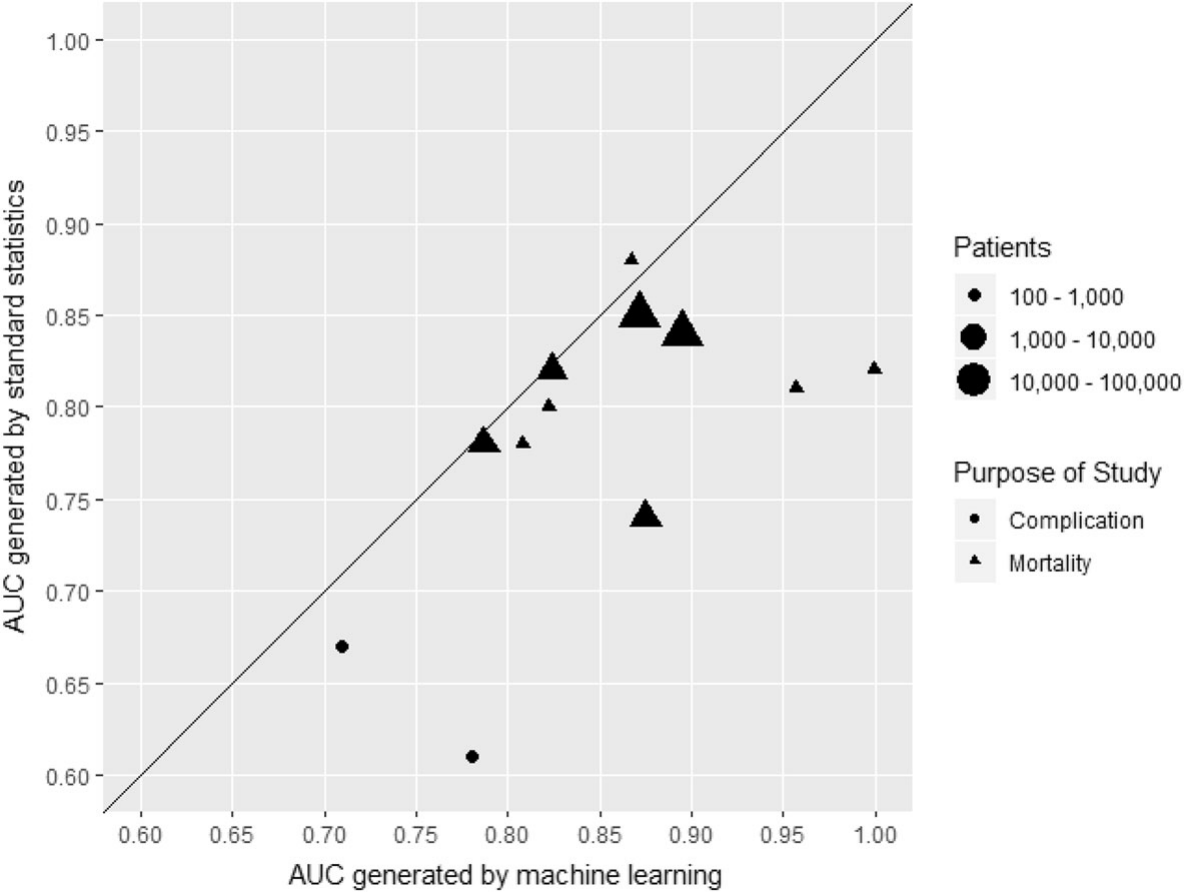
Comparison of AUC scores found in complication or mortality prediction papers according to the technique used to produce them. A line of equality is also provided

Figure 4 shows the distribution of AUC according to the size of dataset, for all prediction studies and for studies predicting mortality or complications, with AUCs from the 10 studies that used external validation shown as individual data points. The median AUC was higher in the smallest studies (< 100 patients) which is likely to reflect over-optimism arising from internal validation in small samples. The median AUC increased with increasing sample size from 100 to 1000 patients to 100,000 to 1,000,000 patients. AUCs for both a machine learning and a standard statistical approach were reported in only 12 studies (Fig. 5). For all but one of these, the machine learning AUC exceeded that from the standard statistical approach. However, the difference appeared related to the study size: three of the four studies with substantial differences between the AUCs were based on fewer than 1000 patients.

The proportion of papers reporting on calibration was low: 30 (11.6%) of the 258 papers included in the review and 23 (13.6%) papers of the 169 studies that predicted complications, mortality, length of stay, or health improvement. Among these 23 papers, 21 reported Hosmer-Lemeshow statistics [21], one reported the Brier score, and one used a graphical approach.

## Discussion

### Key findings

Interest in the use of machine learning to analyse routinely collected ICU data is burgeoning: nearly half of the studies identified in this review were published since 2015. Sample sizes, even in recently reported studies, were often too small to exploit the potential of these methods. Among studies that used machine learning to predict clearly definable outcomes the most commonly used methods were neural networks, support vector machines, and classification/decision trees. Recently reported studies were most likely to use support vector machines, random forests, and neural networks. Most studies validated their predictions using random subsets of the development data, with validations based on independent data rarely reported. Reporting of predictive accuracy was often incomplete, and few studies compared the predictive accuracy of their algorithm with that using standard statistical methods.

### Strengths and limitations

We used comprehensive literature searches but may have omitted studies using proprietary machine learning methods or code repositories that were not peer reviewed and published in the literature databases searched. The study was descriptive, and therefore, the risk of bias was not assessed in the results of included studies. Robust conclusions therefore cannot be drawn about the reasons for the variation in AUC between studies or the differences between the performance of machine learning prediction algorithms and those based on standard statistical techniques. Although there were clear changes in the machine learning techniques used with time, we did not compare the performance of the different techniques. Most of the analyses included in the review related to studies that predicted the clearly definable outcomes of complications, mortality, length of stay, or health improvement: quantitative conclusions about other types of study were not drawn.

### Results in context with literature

The last systematic review of the use of machine learning in the ICU was published in 2001 [9]. It noted the particular suitability of the data-rich ICU environment for machine learning and artificial intelligence. Further narrative reviews stated the need to understand model assumptions and methods to validate predictions when conducting machine learning studies [21, 22]. Papers in this review rarely compared the performance of machine learning with that of predictions derived using standard statistical techniques such as a logistic regression. Empirical studies have suggested that standard statistical techniques produce predictions that are often as accurate as those derived using machine learnings [23]. Standard statistical techniques may have greater transparency with regard to inputs, processing, and outputs: the ‘black-box’ nature of machine learning algorithms can make it difficult to understand the relative importance of the different predictors and the way that they contribute to predictions. This makes it difficult to understand and correct errors when they occur. Thus, studies have highlighted the desirability for transparent reasoning from machine learning algorithms [24]. Our review documents the evolving use of machine learning methods in recent years, but the continuing limitations in the conduct and reporting of the validation of these studies still exist.

Figure 4 suggests that studies based on small sample sizes and using internal validation overestimated model performance. Although there are no fixed minimum dataset sizes appropriate for machine learning applications, data on many tens or hundreds of thousands of patients may be required for these approaches to realize their potential and provide clear advantages over standard statistical analyses [25]. For dichotomous outcomes such as in-hospital mortality, methods such as random forests and support vector machines may demonstrate instability and over-optimism even with more than 200 outcome events per variable [23]. However, the majority of prediction studies included in our review analysed data on fewer than 1000 patients, which is likely to be too few to exploit the power of machine learning [6, 7]. Machine learning techniques are data hungry, and ‘over-fitting’ is more likely in studies based on small sample sizes. Achieving large sample sizes will require continuing development of digital infrastructure that allows linkage between databases and hence generation of datasets on large clinical populations [6, 16, 26]. Truly large datasets (population sizes of > 100,000 individuals) have so far been difficult to generate due to concerns over data privacy and security. Sharing this data with large commercial players who have the programming and processing ability to extract multiple signals from that data is even more difficult [27]. Only three papers included in our review addressed use of machine learning to identify data errors [28–30]. Errors are common in routine EHR data [6], and thus, datasets must be cleaned before analyses. This represents one of the most important tasks in using large datasets and is impossible to do without automation.

### Implications

The most rigorous approach to quantifying the likely performance of machine learning algorithms in future clinical practice, and avoiding over-optimism arising from selection of variables and parametrizations, is to validate algorithms using independent data [31]. However, this was done in only a small minority of studies. Among studies that quantified predictive accuracy, most validated their models using random subsets of their development data. Because patterns of data in such test datasets do not differ systematically from patterns in the training datasets, they may overestimate model performance. A substantial minority of studies did not validate predictions or report the area under the ROC curve. Studies rarely discussed the implementation of machine learning algorithms that had been developed and whether they improved care. They did not report new performance metrics that may overcome limitations of the AUC, such as its insensitivity to the number of false positives when predicting rare events and that it gives equal weight to false positive and false negative predictions [32, 33].

The papers included in our study generally focused on *discrimination* (the ability to differentiate between patients who will and will not experience the outcome). Few studies reported on *calibration* (the degree of agreement between model predictions and the actual outcomes). Model calibration is sensitive to shifts in unmeasured covariates and is particularly important when models are used in population groups that are different from those used for model development.

Reporting standards for applications of machine learning using routinely collected healthcare data, as well as critical appraisal tools, might improve the utility of studies in this area, as has been seen with randomized trials (CONSORT), multivariable prediction models (TRIPOD), and risk of bias in prediction studies (PROBAST) [34–39]. These might assist editors and peer reviewers, for example by avoiding applications based on small datasets and insisting that model performance is evaluated on either an external dataset or, for studies using internal validation, using a separate data subset or procedure appropriate to compensate for statistical over-optimism. To ensure that results are reproducible, and facilitate assessment of discrimination and calibration in new settings, journals and the academic community should promote access to datasets and sharing of analysis code [40].

## Conclusions

The increasing availability of very large and detailed datasets derived from routinely collected ICU data, and widespread recognition of the potential clinical utility of machine learning to develop predictive algorithms based on these data, is leading to rapid increases in the number of studies in this area. However, many published studies are too small to exploit the potential of these methods. Methodological, reporting, and critical appraisal guidelines, particularly with regard to the choice of method and validation of predictions, might increase confidence in reported findings and thereby facilitate the translation of study findings towards routine use in clinical practice.

## Additional files

Additional file 1: Search Terms. The terms used when searching the “Ovid MEDLINE(R) Epub Ahead of Print, In-Process & Other Non-Indexed Citations, Ovid MEDLINE(R) Daily and Ovid MEDLINE(R) 1946 to Present” and “Web of Science” databases. (DOCX 15 kb)

Additional file 2: Full list of papers included in the review. A full reference list of all papers retrieved for full text review. (DOCX 47 kb)

## Data Availability

Dataset used during the current study is available from the corresponding author on request.

## Abbreviations

ICU: Intensive care unit
EHR: Electronic health record
ML: Machine learning
AI: Artificial intelligence
MIMIC-II/III: Medical Information Mart for Intensive Care Version 2 or 3
ROC: Receiver operating characteristic

## References

[1] Xu H, Wu W, Nemati S, Zha H (2017). Patient flow prediction via discriminative learning of mutually-correcting processes. IEEE Trans Knowl Data Eng.

[2] Delahanty RJ, Kaufman D, Jones SS (2018). Development and evaluation of an automated machine learning algorithm for in-hospital mortality risk adjustment among critical care patients. Crit Care Med.

[3] Ruyssinck J, van der Herten J, Houthooft R, Ongenae F, Couckuyt I, Gadeyne B (2016). Random survival forests for predicting the bed occupancy in the intensive care unit. Comput..

[4] Ngufor C, Murphree D, Upadhyaya S, Madde N, Pathak J, Carter R (2016). Predicting prolonged stay in the ICU attributable to bleeding in patients offered plasma transfusion. AMIA Annu Symp Proc.

[5] Ltifi H, Benmohamed E, Kolski C, Ben Ayed M (2016). Enhanced visual data mining process for dynamic decision-making. Knowl-Based Syst.

[6] Johnson AEW, Ghassemi MM, Nemati S, Niehaus KE, Clifton DA, Clifford GD (2016). Machine learning and decision support in critical care. Proc IEEE.

[7] Rajkomar A, Dean J, Kohane I (2019). Machine learning in medicine. N Engl J Med.

[8] Halevy A, Norvig P, Pereira F (2009). The unreasonable effectiveness of data.

[9] Hanson CW, Marshall BE (2001). Artificial intelligence applications in the intensive care unit. Crit Care Med.

[10] Johnson AE, Pollard TJ, Shen L, Li-wei HL, Feng M, Ghassemi M (2016). MIMIC-III, a freely accessible critical care database. Scientific Data.

[11] Moher D, Liberati A, Tetzlaff J, Altman DG, The PRISMA Group (2009). Preferred reporting items for systematic reviews and meta-analyses: the PRISMA statement. PLoS Med.

[12] Davis SE, Lasko TA, Chen G, Siew ED, Matheny ME (2017). Calibration drift in regression and machine learning models for acute kidney injury. J Am Med Inform Assoc.

[13] Koyner JL, Carey KA, Edelson DP, Churpek MM (2018). The development of a machine learning inpatient acute kidney injury prediction model. Crit Care Med.

[14] Liu C-L, Soong R-S, Lee W-C, Chen D-H, Hsu S-H (2018). A predictive model for acute allograft rejection of liver transplantation. Expert Syst Appl.

[15] Liu Y, Traskin M, Lorch SA, George EI, Small D (2015). Ensemble of trees approaches to risk adjustment for evaluating a hospital’s performance. Health Care Manag Sci.

[16] Mao Q, Jay M, Hoffman JL, Calvert J, Barton C, Shimabukuro D (2018). Multicentre validation of a sepsis prediction algorithm using only vital sign data in the emergency department, general ward and ICU. BMJ Open.

[17] Rowan M, Ryan T, Hegarty F, O'Hare N (2007). The use of artificial neural networks to stratify the length of stay of cardiac patients based on preoperative and initial postoperative factors. Artif Intell Med.

[18] Meyfroidt G, Guiza F, Cottem D, De Becker W, Van Loon K, Aerts JM (2011). Computerized prediction of intensive care unit discharge after cardiac surgery: development and validation of a Gaussian processes model. BMC Med Inf Decis Mak..

[19] Tu JV, Guerriere MR (1993). Use of a neural network as a predictive instrument for length of stay in the intensive care unit following cardiac surgery. Comput Biomed Res.

[20] Houthooft R, Ruyssinck J, van der Herten J, Stijven S, Couckuyt I, Gadeyne B (2015). Predictive modelling of survival and length of stay in critically ill patients using sequential organ failure scores. Artif Intell Med.

[21] Barbini E, Cevenini G, Scolletta S, Biagioli B, Giomarelli P, Barbini P (2007). A comparative analysis of predictive models of morbidity in intensive care unit after cardiac surgery - part I: model planning. BMC Med Inf Decis Mak.

[22] Awad A, Bader-El-Den M, McNicholas J (2017). Patient length of stay and mortality prediction: a survey. Health Serv Manag Res.

[23] van der Ploeg T, Austin PC, Steyerberg EW (2014). Modern modelling techniques are data hungry: a simulation study for predicting dichotomous endpoints. BMC Med Res Methodol.

[24] Lisboa PJ (2002). A review of evidence of health benefit from artificial neural networks in medical intervention. Neural Netw.

[25] Beam AL, Kohane IS (2018). Big data and machine learning in health care. Jama..

[26] Kamio T, Van T, Masamune K (2017). Use of machine-learning approaches to predict clinical deterioration in critically ill patients: a systematic review. Int J Med Res Health Sci.

[27] Iacobucci G. Patient data were shared with Google on an “inappropriate legal basis,” says NHS data guardian. BMJ. 2017;357:j2439. 10.1136/bmj.j2439 28522583

[28] de Araujo JM, de Menezes JM, Moura de Albuquerque AA, da Mota Almeida O, Ugulino de Araujo FM (2013). Assessment and certification of neonatal incubator sensors through an inferential neural network. Sensors (Basel).

[29] Huang G, Zhang Y, Cao J, Steyn M, Taraporewalla K (2014). Online mining abnormal period patterns from multiple medical sensor data streams. World Wide Web-Internet Web Information Systems.

[30] Van Loon K, Guiza F, Meyfroidt G, Aerts JM, Ramon J, Blockeel H (2009). Dynamic data analysis and data mining for prediction of clinical stability. Stud Health Technol Inform.

[31] Bailly Sébastien, Meyfroidt Geert, Timsit Jean-François (2017). What’s new in ICU in 2050: big data and machine learning. Intensive Care Medicine.

[32] Hand DJ (2009). Measuring classifier performance: a coherent alternative to the area under the ROC curve. Mach Learn.

[33] Kaymak U, Ben-David A, Potharst R (2012). The AUK: a simple alternative to the AUC. Eng Appl Artif Intell.

[34] Begg C, Cho M, Eastwood S, Horton R, Moher D, Olkin I (1996). Improving the quality of reporting of randomized controlled trials: the CONSORT statement. Jama..

[35] Schulz KF, Altman DG, Moher D (2010). CONSORT 2010 statement: updated guidelines for reporting parallel group randomised trials. BMC Med.

[36] Moher D, Jones A, Lepage L, Group ftC (2001). Use of the CONSORT statement and quality of reports of randomized trials: a comparative before-and-after evaluation. Jama..

[37] Kane RL, Wang J, Garrard J (2007). Reporting in randomized clinical trials improved after adoption of the CONSORT statement. J Clin Epidemiol.

[38] Collins GS, Reitsma JB, Altman DG, Moons KGM (2015). Transparent reporting of a multivariable prediction model for individual prognosis or diagnosis (TRIPOD): the TRIPOD statement. Ann Intern Med.

[39] Wolff RF, Moons KGM, Riley RD, Whiting PF, Westwood M, Collins GS (2019). PROBAST: a tool to assess the risk of bias and applicability of prediction model studies PROBAST (Prediction model Risk Of Bias ASsessment Tool). Ann Intern Med.

[40] Johnson AE, Pollard TJ, Mark RG (2017). Reproducibility in critical care: a mortality prediction case study. Machine Learning for Healthcare Conference.

