# Supplementary material for: Use of machine learning to analyse routinely collected intensive care unit data: a systematic review
Source: Crit Care. 2019 Aug 22;23:284. doi: 10.1186/s13054-019-2564-9 (PMC6704673; doi:10.1186/s13054-019-2564-9)
Supplement: Supplementary file 1 — Search Terms. The terms used when searching the “Ovid MEDLINE(R) Epub Ahead of Print, In-Process & Other Non-Indexed Citations, Ovid MEDLINE(R) Daily and Ovid MEDLINE(R) 1946 to Present” and “Web of Science” databases. (DOCX 15 kb) [file 13054_2019_2564_MOESM1_ESM.docx]

# Additional file 1: Search Terms

**MEDLINE**

We searched the “Ovid MEDLINE(R) Epub Ahead of Print, In-Process & Other Non-Indexed Citations, Ovid MEDLINE(R) Daily and Ovid MEDLINE(R) 1946 to Present” databases.

1. Artificial Intelligence/ or Machine Learning/
2. Neural Networks Computer/
3. Support Vector Machine/
4. Models, theoretical/ or fuzzy logic/ or models, organizational/
5. Data mining/
6. Pattern Recognition, Automated/
7. Machine learning.tw
8. Artificial intelligence.tw
9. Deep learning.tw
10. Neural network.tw
11. Support vector machine?.tw
12. Prediction network.tw
13. Forecast model*.tw
14. Data mining.tw
15. Supervised learning.tw
16. Time series prediction.tw
17. Patient Flow.tw
18. Length of Stay.tw
19. Demand forecasting.tw
20. Mortality Prediction.tw
21. Operational Forecasting.tw
22. 1 or 2 or 3 or 4 or 5 or 6 or 7 or 8 or 9 or 10 or 11 or 12 or 13 or 14 or 15 or 16 or 17 or 19 or 20 or 21
23. Critical Care/
24. Intensive care units/ or coronary care units/
25. High dependency unit.tw
26. Cardiac Intensive Care Unit.tw
27. High dependency unit.tw
28. ICU.tw
29. ?ICU.tw
30. PICU.tw
31. Intensive Care Unit.tw
32. Intensive care.tw
33. Coronary care.tw
34. Critical Care.tw
35. 23 or 24 or 25 or 26 or 27 or 28 or 29 or 30 or 31 or 32 or 33 or 34
36. 22 and 35

**1. Artificial Intelligence/ or Machine Learning/**

**2. Neural Networks Computer/**

**3. Support Vector Machine/**

**4. fuzzy logic/**

**5. Data mining/**

**6. Pattern Recognition, Automated/**

**7. machine learning.tw.**

**8. artificial intelligence.tw.**

**9. deep learning.tw.**

**10. neural network.tw.**

**11. support vector machine?.tw.**

**12. prediction network.tw.**

**13. forecast model*.tw.**

**14. Data mining.tw.**

**15. Supervised Learning.tw.**

**16. Time Series Prediction.tw.**

**17. Patient Flow.tw.**

**18. Length of stay.tw.**

**19. Demand Forecasting.tw.**

**20. Mortality Prediction.tw.**

**21. Operational Forecasting.tw.**

**22. 1 or 2 or 3 or 4 or 5 or 6 or 7 or 8 or 9 or 10 or 11 or 12 or 13 or 14 or 15 or 16 or 17 or 19 or 20 or 21**

**23. Critical Care/**

**24. intensive care units/ or coronary care units/**

**25. High Dependency Unit.tw.**

**26. ICU.tw.**

**27. PICU.tw.**

**28. NICU.tw.**

**29. GICU.tw.**

**30. CICU.tw.**

**31. NCCU.tw.**

**32. Intensive Care Unit.tw.**

**33. Intensive Care.tw.**

**34. Coronary Care.tw.**

**35. Critical Care.tw.**

**36. 23 or 24 or 25 or 26 or 27 or 28 or 29 or 30 or 31 or 32 or 33 or 34 or 35**

**37. 22 and 36**

**Web of Science**

All databases within the Web of Science site were used.

("Machine Learning" OR "Artificial Intelligence" OR "Deep Learning" OR "Neural Network" OR "Support vector machin*" OR "Prediction Network" OR "Forecast Mode*" OR "Data mining" OR "Supervised Learning" OR "Time series prediction") AND TOPIC: ("Cardiac Intensive Care Unit" OR "CICU" OR "ICU" OR "Coronary Care" OR "Critical Care" OR "High Dependency" OR "HDU")
