# Supplementary material for: Use of machine learning to analyse routinely collected intensive care unit data: a systematic review
Source: Crit Care. 2019 Aug 22;23:284. doi: 10.1186/s13054-019-2564-9 (PMC6704673; doi:10.1186/s13054-019-2564-9)
Supplement: Supplementary file 2 — Full list of papers included in the review. A full reference list of all papers retrieved for full text review. (DOCX 47 kb) [file 13054_2019_2564_MOESM2_ESM.docx]

# Additional file 2: Full list of papers included in the review

1. Aarabi A, Grebe R, Wallois F. A multistage knowledge-based system for EEG seizure detection in newborn infants. Clin Neurophysiol. 2007;118(12):2781-97.

2. Abu-Hanna A, de Keizer N. Integrating classification trees with local logistic regression in Intensive Care prognosis. Artificial Intelligence in Medicine. 2003;29(1-2):5-23.

3. Adlassnig KP, Blacky A, Koller W. Artificial-intelligence-based hospital-acquired infection control. Stud Health Technol Inform. 2009;149:103-10.

4. Alderden J, Pepper GA, Wilson A, Whitney JD, Richardson S, Butcher R, et al. Predicting Pressure Injury in Critical Care Patients: A Machine-Learning Model. American journal of critical care : an official publication, American Association of Critical-Care Nurses. 2018;27(6):461-8.

5. Ambalavanan N, Nelson KG, Alexander G, Johnson SE, Biasini F, Carlo WA. Prediction of neurologic morbidity in extremely low birth weight infants. J Perinatol. 2000;20(8 Pt 1):496-503.

6. Anand RS, Stey P, Jain S, Biron DR, Bhatt H, Monteiro K, et al. Predicting Mortality in Diabetic ICU Patients Using Machine Learning and Severity Indices. AMIA Joint Summits on Translational Science proceedings AMIA Joint Summits on Translational Science. 2018;2017:310-9.

7. Anderson GT, Zheng U, Wyeth R, Johnson A, Bissett J, Grp P. A rough set/fuzzy logic based decision making system for medical applications. International Journal of General Systems. 2000;29(6):879-96.

8. Ansari AH, Matic V, De Vos M, Naulaers G, Cherian PJ, Van Huffel S. Improvement of an automated neonatal seizure detector using a post-processing technique. Conf Proc IEEE Eng Med Biol Soc. 2015;2015:5859-62.

9. Ansari S, Belle A, Ghanbari H, Salamango M, Najarian K. Suppression of false arrhythmia alarms in the ICU: a machine learning approach. Physiological Measurement. 2016;37(8):1186-203.

10. Antink CH, Leonhardt S, Walter M. Reducing false alarms in the ICU by quantifying self-similarity of multimodal biosignals. Physiological Measurement. 2016;37(8):1233-52.

11. Aoki N, Wall MJ, Demsar J, Zupan B, Granchi T, Schreiber MA, et al. Predictive model for survival at the conclusion of a damage control laparotomy. Am J Surg. 2000;180(6):540-4; discussion 4-5.

12. Arizmendi C, Viviescas J, Gonzalez H, Giraldo B. Patients classification on weaning trials using neural networks and wavelet transform. Stud Health Technol Inform. 2014;202:107-10.

13. Ashutosh K, Lee H, Mohan CK, Ranka S, Mehrotra K, Alexander C. Prediction criteria for successful weaning from respiratory support: statistical and connectionist analyses.[Erratum appears in Crit Care Med. 1994 Jan;22(1):183; PMID: 8124967]. Critical Care Medicine. 1992;20(9):1295-301.

14. Aushev A, Ripoll VR, Vellido A, Aletti F, Pinto BB, Herpain A, et al. Feature selection for the accurate prediction of septic and cardiogenic shock ICU mortality in the acute phase. Plos One. 2018;13(11).

15. Awad A, Bader-El-Den M, McNicholas J, Briggs J. Early hospital mortality prediction of intensive care unit patients using an ensemble learning approach. International Journal of Medical Informatics. 2017;108:185-95.

16. Bai Y, Do DH, Harris PR, Schindler D, Boyle NG, Drew BJ, et al. Integrating monitor alarms with laboratory test results to enhance patient deterioration prediction. Journal of Biomedical Informatics. 2015;53:81-92.

17. Banner MJ, Euliano NR, Brennan V, Peters C, Layon AJ, Gabrielli A. Power of breathing determined noninvasively with use of an artificial neural network in patients with respiratory failure. Critical Care Medicine. 2006;34(4):1052-9.

18. Barro S, Marin R, Palacios F, Ruiz R. Fuzzy logic in a patient supervision system. Artificial Intelligence in Medicine. 2001;21(1-3):193-9.

19. Bataille B, Riu B, Ferre F, Moussot PE, Mari A, Brunel E, et al. Integrated use of bedside lung ultrasound and echocardiography in acute respiratory failure: a prospective observational study in ICU. Chest. 2014;146(6):1586-93.

20. Baumgartner B, Rodel K, Knoll A. A data mining approach to reduce the false alarm rate of patient monitors. Conf Proc IEEE Eng Med Biol Soc. 2012;2012:5935-8.

21. Baxt WG. A NEURAL-NETWORK TRAINED TO IDENTIFY THE PRESENCE OF MYOCARDIAL-INFARCTION BASES SOME DECISIONS ON CLINICAL ASSOCIATIONS THAT DIFFER FROM ACCEPTED CLINICAL TEACHING. Medical Decision Making. 1994;14(3):217-22.

22. Beaulieu-Jones BK, Orzechowski P, Moore JH. Mapping Patient Trajectories using Longitudinal Extraction and Deep Learning in the MIMIC-III Critical Care Database. Pac Symp Biocomput. 2018;23:123-32.

23. Behar J, Oster J, Li Q, Clifford GD. ECG signal quality during arrhythmia and its application to false alarm reduction. IEEE Transactions on Biomedical Engineering. 2013;60(6):1660-6.

24. Belal SY, Emmerson AJ, Beatty PC. Automatic detection of apnoea of prematurity. Physiological Measurement. 2011;32(5):523-42.

25. Belal SY, Taktak AF, Nevill A, Spencer A. An intelligent ventilation and oxygenation management system in neonatal intensive care using fuzzy trend template fitting. Physiological Measurement. 2005;26(4):555-70.

26. Belderrar A, Hazzab A. Hierarchical Genetic Algorithm and Fuzzy Radial Basis Function Networks for Factors Influencing Hospital Length of Stay Outliers. Healthcare Informatics Research. 2017;23(3):226-32.

27. Bogaarts JG, Hilkman DMW, Gommer ED, van Kranen-Mastenbroek VHJM, Reulen JPH. Improved epileptic seizure detection combining dynamic feature normalization with EEG novelty detection. Medical & Biological Engineering & Computing. 2016;54(12):1883-92.

28. Braga A, Portela F, Santos MF, Abelha A, Machado J, Silva A, et al. DATA MINING TO PREDICT THE USE OF VASOPRESSORS IN INTENSIVE MEDICINE PATIENTS. Jurnal Teknologi. 2016;78(6-7):1-6.

29. Buchman TG, Kubos KL, Seidler AJ, Siegforth MJ. A COMPARISON OF STATISTICAL AND CONNECTIONIST MODELS FOR THE PREDICTION OF CHRONICITY IN A SURGICAL INTENSIVE-CARE UNIT. Critical Care Medicine. 1994;22(5):750-62.

30. Calvert J, Mao Q, Hoffman JL, Jay M, Desautels T, Mohamadlou H, et al. Using electronic health record collected clinical variables to predict medical intensive care unit mortality. Ann Med Surg (Lond). 2016;11:52-7.

31. Calvert JS, Price DA, Chettipally UK, Barton CW, Feldman MD, Hoffman JL, et al. A computational approach to early sepsis detection. Computers in Biology and Medicine. 2016;74:69-73.

32. Carlin CS, Ho LV, Ledbetter DR, Aczon MD, Wetzel RC. Predicting individual physiologically acceptable states at discharge from a pediatric intensive care unit. Journal of the American Medical Informatics Association. 2018;06:06.

33. Celi LA, Galvin S, Davidzon G, Lee J, Scott D, Mark R. A Database-driven Decision Support System: Customized Mortality Prediction. Journal of Personalized Medicine. 2012;2(4):138-48.

34. Celi LA, Hinske LC, Alterovitz G, Szolovits P. An artificial intelligence tool to predict fluid requirement in the intensive care unit: a proof-of-concept study. Crit Care. 2008;12(6):R151.

35. Chan CH, Chan EY, Ng DK, Chow PY, Kwok KL. Application of artificial neural networks to establish a predictive mortality risk model in children admitted to a paediatric intensive care unit. Singapore Med J. 2006;47(11):928-34.

36. Chaparro JA, Giraldo BF, Caminal P, Benito S. Performance of respiratory pattern parameters in classifiers for predict weaning process. Conf Proc IEEE Eng Med Biol Soc. 2012;2012:4349-52.

37. Chaves LE, Nascimento LF. Estimating outcomes in newborn infants using fuzzy logic. Revista Paulista de Pediatria. 2014;32(2):164-70.

38. Che Z, Purushotham S, Khemani R, Liu Y. Interpretable Deep Models for ICU Outcome Prediction. AMIA Annu Symp Proc. 2016;2016:371-80.

39. Chen W, Wang Y, Cao G, Chen G, Gu Q. A random forest model based classification scheme for neonatal amplitude-integrated EEG. Biomedical Engineering Online. 2014;13 Suppl 2:S4.

40. Chen Z, Bird VY, Ruchi R, Segal MS, Bian J, Khan SR, et al. Development of a personalized diagnostic model for kidney stone disease tailored to acute care by integrating large clinical, demographics and laboratory data: the diagnostic acute care algorithm - kidney stones (DACA-KS). Bmc Medical Informatics and Decision Making. 2018;18.

41. Chien S-W, Bau C-T, Lin K-C, Wang K-A, Chen Y-F, Chen J-C. EVALUATION OF INTELLIGENT SYSTEM TO THE CONTROL OF DIABETES. International Journal of Innovative Computing Information and Control. 2012;8(1B):779-90.

42. Cho I, Park I, Kim E, Lee E, Bates DW. Using EHR data to predict hospital-acquired pressure ulcers: a prospective study of a Bayesian Network model. International Journal of Medical Informatics. 2013;82(11):1059-67.

43. Chouvarda IG, Babalis D, Papaioannou V, Maglaveras N, Georgopoulos D. Multiparametric modeling of the ineffective efforts in assisted ventilation within an ICU. Medical & Biological Engineering & Computing. 2016;54(2-3):441-51.

44. Christov I, Bortolan G. Ranking of pattern recognition parameters for premature ventricular contractions classification by neural networks. Physiological Measurement. 2004;25(5):1281-90.

45. Cismondi F, Celi LA, Fialho AS, Vieira SM, Reti SR, Sousa JM, et al. Reducing unnecessary lab testing in the ICU with artificial intelligence. International Journal of Medical Informatics. 2013;82(5):345-58.

46. Conroy B, Eshelman L, Potes C, Xu-Wilson M. A dynamic ensemble approach to robust classification in the presence of missing data. Machine Learning. 2016;102(3):443-63.

47. Davis SE, Lasko TA, Chen G, Siew ED, Matheny ME. Calibration drift in regression and machine learning models for acute kidney injury. Journal of the American Medical Informatics Association. 2017;24(6):1052-61.

48. Davoodi R, Moradi MH. Mortality prediction in intensive care units (ICUs) using a deep rule-based fuzzy classifier. Journal of Biomedical Informatics. 2018;79:48-59.

49. de Araujo JM, de Menezes JM, Moura de Albuquerque AA, da Mota Almeida O, Ugulino de Araujo FM. Assessment and certification of neonatal incubator sensors through an inferential neural network. Sensors (Basel). 2013;13(11):15613-32.

50. De Pasquale M, Moss TJ, Cerutti S, Calland JF, Lake DE, Moorman JR, et al. Hemorrhage Prediction Models in Surgical Intensive Care: Bedside Monitoring Data Adds Information to Lab Values. IEEE j. 2017;21(6):1703-10.

51. Delahanty RJ, Kaufman D, Jones SS. Development and Evaluation of an Automated Machine Learning Algorithm for In-Hospital Mortality Risk Adjustment Among Critical Care Patients. Critical Care Medicine. 2018;06:06.

52. Demsar J, Zupan B, Aoki N, Wall MJ, Granchi TH, Beck JR. Feature mining and predictive model construction from severe trauma patient's data. International Journal of Medical Informatics. 2001;63(1-2):41-50.

53. Dervishi A. Fuzzy risk stratification and risk assessment model for clinical monitoring in the ICU. Comput Biol Med. 2017;87:169-78.

54. Desautels T, Calvert J, Hoffman J, Jay M, Kerem Y, Shieh L, et al. Prediction of Sepsis in the Intensive Care Unit With Minimal Electronic Health Record Data: A Machine Learning Approach. JMIR Medical Informatics. 2016;4(3):e28.

55. Desautels T, Das R, Calvert J, Trivedi M, Summers C, Wales DJ, et al. Prediction of early unplanned intensive care unit readmission in a UK tertiary care hospital: a cross-sectional machine learning approach. Bmj Open. 2017;7(9).

56. Dickerson RN, Mason DL, Croce MA, Minard G, Brown RO. Evaluation of an artificial neural network to predict urea nitrogen appearance for critically ill multiple-trauma patients. JPEN J Parenter Enteral Nutr. 2005;29(6):429-35.

57. Ding Y, Li X, Wang Y, Ieee. Mortality Prediction for ICU Patients Using Just-in-time Learning and Extreme Learning Machine2016. 939-44 p.

58. Ding Y, Ma X, Wang Y. Health status monitoring for ICU patients based on locally weighted principal component analysis. Computer Methods and Programs in Biomedicine. 2018;156:61-71.

59. Ding Y, Wang Y, Zhou D. Mortality prediction for ICU patients combining just-in-time learning and extreme learning machine. Neurocomputing. 2018;281:12-9.

60. Doan TN, Kong DC, Marshall C, Kirkpatrick CM, McBryde ES. Characterising the Transmission Dynamics of Acinetobacter baumannii in Intensive Care Units Using Hidden Markov Models. PLoS ONE [Electronic Resource]. 2015;10(7):e0132037.

61. Doig GS, Inman KJ, Sibbald WJ, Martin CM, Robertson JM. Modeling mortality in the intensive care unit: comparing the performance of a back-propagation, associative-learning neural network with multivariate logistic regression. Proc Annu Symp Comput Appl Med Care. 1993:361-5.

62. Donald R, Howells T, Piper I, Chambers I, Citerio G, Enblad P, et al. Early warning of EUSIG-defined hypotensive events using a Bayesian Artificial Neural Network. Acta Neurochir Suppl. 2012;114:39-44.

63. Donald R, Howells T, Piper I, Enblad P, Nilsson P, Chambers I, et al. Forewarning of hypotensive events using a Bayesian artificial neural network in neurocritical care. Journal of clinical monitoring and computing. 2018.

64. Downs J, Harrison RF, Kennedy RL, Cross SS. Application of the fuzzy ARTMAP neural network model to medical pattern classification tasks. Artificial Intelligence in Medicine. 1996;8(4):403-28.

65. Eerikainen LM, Vanschoren J, Rooijakkers MJ, Vullings R, Aarts RM. Reduction of false arrhythmia alarms using signal selection and machine learning. Physiological Measurement. 2016;37(8):1204-16.

66. Ennett CM, Frize M, Charette E. Improvement and automation of artificial neural networks to estimate medical outcomes. Medical Engineering & Physics. 2004;26(4):321-8.

67. Ennett CM, Frize M, Walker CR. Influence of missing values on artificial neural network performance. Stud Health Technol Inform. 2001;84(Pt 1):449-53.

68. Enright CG, Madden MG, Madden N. Bayesian networks for mathematical models: Techniques for automatic construction and efficient inference. International Journal of Approximate Reasoning. 2013;54(2):323-42.

69. Fadlalla AM, Golob JF, Jr., Claridge JA. Enhancing the fever workup utilizing a multi-technique modeling approach to diagnose infections more accurately. Surg Infect (Larchmt). 2012;13(2):93-101.

70. Fialho AS, Cismondi F, Vieira SM, Reti SR, Sousa JMC, Finkelstein SN. Data mining using clinical physiology at discharge to predict ICU readmissions. Expert Systems with Applications. 2012;39(18):13158-65.

71. Flanagan JR, Pittet D, Li N, Thievent B, Suter PM, Wenzel RP. Predicting survival of patients with sepsis by use of regression and neural network models. Clin Perform Qual Health Care. 1996;4(2):96-103.

72. Foltran F, Berchialla P, Giunta F, Malacarne P, Merletti F, Gregori D. Using VLAD scores to have a look insight ICU performance: towards a modelling of the errors. Journal of Evaluation in Clinical Practice. 2010;16(5):968-75.

73. Fong A, Mittu R, Ratwani R, Reggia J. Predicting electrocardiogram and arterial blood pressure waveforms with different Echo State Network architectures. AMIA Annu Symp Proc. 2014;2014:544-53.

74. Frize M, Walker RC, Ibrahim D. Identifying risk factors for two complication types for neonatal intensive care patients (NICU). Conf Proc IEEE Eng Med Biol Soc. 2006;1:2324-7.

75. Frize M, Wang L, Ennett CM, Nickerson BG, Solven FG, Stevenson M. New advances and validation of knowledge management tools for critical care using classifier techniques. Proceedings AMIA Symposium. 1998:553-7.

76. Fujita H, Acharya UR, Sudarshan VK, Ghista DN, Sree SV, Eugene LWJ, et al. Sudden cardiac death (SCD) prediction based on nonlinear heart rate variability features and SCD index. Applied Soft Computing. 2016;43:510-9.

77. Ganzert S, Guttmann J, Kersting K, Kuhlen R, Putensen C, Sydow M, et al. Analysis of respiratory pressure-volume curves in intensive care medicine using inductive machine learning. Artificial Intelligence in Medicine. 2002;26(1-2):69-86.

78. Garde A, Schroeder R, Voss A, Caminal P, Benito S, Giraldo BF. Patients on weaning trials classified with support vector machines. Physiological Measurement. 2010;31(7):979-93.

79. Gholami B, Phan TS, Haddad WM, Cason A, Mullis J, Price L, et al. Replicating human expertise of mechanical ventilation waveform analysis in detecting patient-ventilator cycling asynchrony using machine learning. Computers in Biology and Medicine. 2018;97:137-44.

80. Gholipour C, Rahim F, Fakhree A, Ziapour B. Using an Artificial Neural Networks (ANNs) Model for Prediction of Intensive Care Unit (ICU) Outcome and Length of Stay at Hospital in Traumatic Patients. J Clin Diagn Res. 2015;9(4):OC19-23.

81. Ghose S, Mitra J, Khanna S, Dowling J. An Improved Patient-Specific Mortality Risk Prediction in ICU in a Random Forest Classification Framework. Stud Health Technol Inform. 2015;214:56-61.

82. Ghosh S, Feng M, Nguyen H, Li J. Risk prediction for acute hypotensive patients by using gap constrained sequential contrast patterns. AMIA Annual Symposium proceedings AMIA Symposium. 2014;2014:1748-57.

83. Ghosh S, Feng M, Nguyen H, Li J. Hypotension Risk Prediction via Sequential Contrast Patterns of ICU Blood Pressure. IEEE journal of biomedical and health informatics. 2016;20(5):1416-26.

84. Ghosh S, Hung N, Jinyan L. Predicting short-term ICU outcomes using a sequential contrast motif based classification framework. Conf Proc IEEE Eng Med Biol Soc. 2016;2016:5612-5.

85. Ghosh S, Li J, Cao L, Ramamohanarao K. Septic shock prediction for ICU patients via coupled HMM walking on sequential contrast patterns. Journal of Biomedical Informatics. 2017;66:19-31.

86. Giraldo B, Arizmendi C, Romero E, Alquezar R, Caminal P, Benito S, et al. Patients on weaning trials from mechanical ventilation classified with neural networks and feature selection. Conf Proc IEEE Eng Med Biol Soc. 2006;1:2195-8.

87. Giraldo BF, Chaparro JA, Caminal P, Benito S. Characterization of the respiratory pattern variability of patients with different pressure support levels. Conf Proc IEEE Eng Med Biol Soc. 2013;2013:3849-52.

88. Gonzalez-Robledo J, Martin-Gonzalez F, Sanchez-Barba M, Sanchez-Hernandez F, Moreno-Garcia MN. Multiclassifier Systems for Predicting Neurological Outcome of Patients with Severe Trauma and Polytrauma in Intensive Care Units. Journal of Medical Systems. 2017;41(9).

89. Gortzis LG, Sakellaropoulos F, Ilias I, Stamoulis K, Dimopoulou I. Predicting ICU survival: a meta-level approach. BMC Health Services Research. 2008;8:157.

90. Goss EP, Vozikis GS. Improving health care organizational management through neural network learning. Health care management science. 2002;5(3):221-7.

91. Guiza F, Depreitere B, Piper I, Van den Berghe G, Meyfroidt G. Novel methods to predict increased intracranial pressure during intensive care and long-term neurologic outcome after traumatic brain injury: development and validation in a multicenter dataset. Critical Care Medicine. 2013;41(2):554-64.

92. Hanisch E, Brause R, Paetz J, Arlt B. Review of a large clinical series: Predicting death for patients with abdominal septic shock. Journal of Intensive Care Medicine. 2011;26(1):27-33.

93. Hao D, Ghassemi MM, Mengling F. The effects of deep network topology on mortality prediction. Conf Proc IEEE Eng Med Biol Soc. 2016;2016:2602-5.

94. Hoogendoorn M, El Hassouni A, Mok K, Ghassemi M, Szolovits P. Prediction using patient comparison vs. modeling: a case study for mortality prediction. Conf Proc IEEE Eng Med Biol Soc. 2016;2016:2464-7.

95. Houthooft R, Ruyssinck J, van der Herten J, Stijven S, Couckuyt I, Gadeyne B, et al. Predictive modelling of survival and length of stay in critically ill patients using sequential organ failure scores. Artificial Intelligence in Medicine. 2015;63(3):191-207.

96. Hsieh MH, Hsieh MJ, Chen CM, Hsieh CC, Chao CM, Lai CC. An Artificial Neural Network Model for Predicting Successful Extubation in Intensive Care Units. Journal of Clinical Medicine. 2018;7(9):25.

97. Hsieh MH, Hsieh MJ, Chen C-M, Hsieh C-C, Chao C-M, Lai C-C. Comparison of machine learning models for the prediction of mortality of patients unplanned extubation in intensive with care units. Scientific Reports. 2018;8.

98. Hsieh YZ, Su MC, Wang CH, Wang PC. Prediction of survival of ICU patients using computational intelligence. Comput Biol Med. 2014;47:13-9.

99. Hsu CC, Lin YE, Chen YS, Liu YC, Muder RR. Validation study of artificial neural network models for prediction of methicillin-resistant Staphylococcus aureus carriage. Infect Control Hosp Epidemiol. 2008;29(7):607-14.

100. Hu SB, Wong DJ, Correa A, Li N, Deng JC. Prediction of Clinical Deterioration in Hospitalized Adult Patients with Hematologic Malignancies Using a Neural Network Model. PLoS ONE [Electronic Resource]. 2016;11(8):e0161401.

101. Huang G, Zhang Y, Cao J, Steyn M, Taraporewalla K. Online mining abnormal period patterns from multiple medical sensor data streams. World Wide Web-Internet and Web Information Systems. 2014;17(4):569-87.

102. Huddar V, Desiraju BK, Rajan V, Bhattacharya S, Roy S, Reddy CK. Predicting Complications in Critical Care Using Heterogeneous Clinical Data. Ieee Access. 2016;4:7988-8001.

103. Irles C, Gonzalez-Perez G, Carrera Muinos S, Michel Macias C, Sanchez Gomez C, Martinez-Zepeda A, et al. Estimation of Neonatal Intestinal Perforation Associated with Necrotizing Enterocolitis by Machine Learning Reveals New Key Factors. Int J Environ Res Public Health. 2018;15(11):09.

104. Izenberg SD, Williams MD, Luterman A. Prediction of trauma mortality using a neural network. Am Surg. 1997;63(3):275-81.

105. Jalali A, Bender D, Rehman M, Nadkanri V, Nataraj C. Advanced analytics for outcome prediction in intensive care units. Conf Proc IEEE Eng Med Biol Soc. 2016;2016:2520-4.

106. Ji SY, Smith R, Huynh T, Najarian K. A comparative analysis of multi-level computer-assisted decision making systems for traumatic injuries. BMC Med Inf Decis Mak. 2009;9:2.

107. Jimenez F, Sanchez G, Juarez JM. Multi-objective evolutionary algorithms for fuzzy classification in survival prediction. Artificial Intelligence in Medicine. 2014;60(3):197-219.

108. Johnson AEW, Kramer AA, Clifford GD. A New Severity of Illness Scale Using a Subset of Acute Physiology and Chronic Health Evaluation Data Elements Shows Comparable Predictive Accuracy. Critical Care Medicine. 2013;41(7):1711-8.

109. Johnson AEW, Mark RG. Real-time mortality prediction in the Intensive Care Unit. AMIA Annu Symp Proc. 2017;2017:994-1003.

110. Joshi R, Szolovits P. Prognostic physiology: modeling patient severity in Intensive Care Units using radial domain folding. AMIA Annual Symposium proceedings AMIA Symposium. 2012;2012:1276-83.

111. Kaewprag P, Newton C, Vermillion B, Hyun S, Huang K, Machiraju R. Predictive Modeling for Pressure Ulcers from Intensive Care Unit Electronic Health Records. AMIA Joint Summits on Translational Science proceedings AMIA Joint Summits on Translational Science. 2015;2015:82-6.

112. Kale DC, Che Z, Bahadori MT, Li W, Liu Y, Wetzel R. Causal Phenotype Discovery via Deep Networks. AMIA Annu Symp Proc. 2015;2015:677-86.

113. Kalidas V, Tamil LS. Cardiac arrhythmia classification using multi-modal signal analysis. Physiological Measurement. 2016;37(8):1253-72.

114. Kam HJ, Kim HY. Learning representations for the early detection of sepsis with deep neural networks. Comput Biol Med. 2017;89:248-55.

115. Kamaleswaran R, Akbilgic O, Hallman MA, West AN, Davis RL, Shah SH. Applying Artificial Intelligence to Identify Physiomarkers Predicting Severe Sepsis in the PICU. Pediatric Critical Care Medicine. 2018;19(10):e495-e503.

116. Kamio T, Van T, Masamune K. Use of Machine-Learning Approaches to Predict Clinical Deterioration in Critically Ill Patients: A Systematic Review. International Journal of Medical Research & Health Sciences. 2017;6(6):1-7.

117. Kannathal N, Acharya UR, Lim CM, Sadasivan P, Krishnan S. Classification of cardiac patient states using artificial neural networks. Exp. 2003;8(4):206-11.

118. Kayaalp M, Cooper GF, Clermont G. Predicting ICU mortality: a comparison of stationary and nonstationary temporal models. Proc AMIA Symp. 2000;Annual Symposium.:418-22.

119. Kennedy CE, Aoki N, Mariscalco M, Turley JP. Using Time Series Analysis to Predict Cardiac Arrest in a PICU. Pediatric Critical Care Medicine. 2015;16(9):e332-9.

120. Kilic YA, Konan A, Yorganci K, Sayek I. A novel fuzzy-logic inference system for predicting trauma-related mortality: emphasis on the impact of response to resuscitation. Eur. 2010;36(6):543-50.

121. Kim S, Kim W, Park RW. A Comparison of Intensive Care Unit Mortality Prediction Models through the Use of Data Mining Techniques. Healthcare Informatics Research. 2011;17(4):232-43.

122. Kim S-H, Li L, Faloutsos C, Yang H-J, Lee S-W. HeartCast: Predicting acute hypotensive episodes in intensive care units. Statistical Methodology. 2016;33:1-13.

123. Koetsier A, de Keizer NF, Abu-Hanna A, Peek N. A modified real AdaBoost algorithm to discover intensive care unit subgroups with a poor outcome. AMIA Annual Symposium proceedings AMIA Symposium. 2013;2013:798-803.

124. Komorowski M, Celi LA, Badawi O, Gordon AC, Faisal AA. The Artificial Intelligence Clinician learns optimal treatment strategies for sepsis in intensive care. Nat Med. 2018;24(11):1716-20.

125. Kong G, Xu D-L, Yang J-B, Yin X, Wang T, Jiang B, et al. Belief rule-based inference for predicting trauma outcome. Knowledge-Based Systems. 2016;95:35-44.

126. Koolen N, Oberdorfer L, Rona Z, Giordano V, Werther T, Klebermass-Schrehof K, et al. Automated classification of neonatal sleep states using EEG. Clin Neurophysiol. 2017;128(6):1100-8.

127. Koyner JL, Carey KA, Edelson DP, Churpek MM. The Development of a Machine Learning Inpatient Acute Kidney Injury Prediction Model. Critical Care Medicine. 2018;46(7):1070-7.

128. Kreif N, Grieve R, Diaz I, Harrison D. Evaluation of the Effect of a Continuous Treatment: A Machine Learning Approach with an Application to Treatment for Traumatic Brain Injury. Health Economics. 2015;24(9):1213-28.

129. Kreif N, Tran L, Grieve R, De Stavola B, Tasker RC, Petersen M. Estimating the Comparative Effectiveness of Feeding Interventions in the Pediatric Intensive Care Unit: A Demonstration of Longitudinal Targeted Maximum Likelihood Estimation. Am J Epidemiol. 2017;186(12):1370-9.

130. Kuo HJ, Chiu HW, Lee CN, Chen TT, Chang CC, Bien MY. Improvement in the Prediction of Ventilator Weaning Outcomes by an Artificial Neural Network in a Medical ICU. Respiratory Care. 2015;60(11):1560-9.

131. Kusiak A, Caldarone CA, Kelleher MD, Lamb FS, Persoon TJ, Burns A. Hypoplastic left heart syndrome: knowledge discovery with a data mining approach. Computers in Biology and Medicine. 2006;36(1):21-40.

132. LaFaro RJ, Pothula S, Kubal KP, Inchiosa ME, Pothula VM, Yuan SC, et al. Neural Network Prediction of ICU Length of Stay Following Cardiac Surgery Based on Pre-Incision Variables. PLoS ONE [Electronic Resource]. 2015;10(12):e0145395.

133. Lamping F, Jack T, Rubsamen N, Sasse M, Beerbaum P, Mikolajczyk RT, et al. Development and validation of a diagnostic model for early differentiation of sepsis and non-infectious SIRS in critically ill children - a data-driven approach using machine-learning algorithms. BMC Pediatr. 2018;18(1):112.

134. Lee H, Shin SY, Seo M, Nam GB, Joo S. Prediction of Ventricular Tachycardia One Hour before Occurrence Using Artificial Neural Networks. Scientific Reports. 2016;6:32390.

135. Lee J. Patient-Specific Predictive Modeling Using Random Forests: An Observational Study for the Critically Ill. JMIR Medical Informatics. 2017;5(1):e3.

136. Lee J, Mark R. A Hypotensive Episode Predictor for Intensive Care based on Heart Rate and Blood Pressure Time Series. Computing in Cardiology. 2011;2010(26-29 Sept. 2010):81-4.

137. Lee J, Mark RG. An investigation of patterns in hemodynamic data indicative of impending hypotension in intensive care. Biomedical Engineering Online. 2010;9:62.

138. Legrand M, Pirracchio R, Rosa A, Petersen ML, Van der Laan M, Fabiani JN, et al. Incidence, risk factors and prediction of post-operative acute kidney injury following cardiac surgery for active infective endocarditis: an observational study. Crit Care. 2013;17(5):R220.

139. Lehman LW, Mark R, Nemati S. A Model-based Machine Learning Approach to Probing Autonomic Regulation from Nonstationary Vital-Signs Time Series. IEEE j. 2016;07:07.

140. Lehman LW, Nemati S, Adams RP, Mark RG. Discovering shared dynamics in physiological signals: application to patient monitoring in ICU. Conf Proc IEEE Eng Med Biol Soc. 2012;2012:5939-42.

141. Leite CR, Sizilio GR, Neto AD, Valentim RA, Guerreiro AM. A fuzzy model for processing and monitoring vital signs in ICU patients. Biomedical Engineering Online. 2011;10:68.

142. Li Q, Clifford GD. Signal quality and data fusion for false alarm reduction in the intensive care unit. Journal of Electrocardiology. 2012;45(6):596-603.

143. Li Q, Clifford GD. Dynamic time warping and machine learning for signal quality assessment of pulsatile signals. Physiological Measurement. 2012;33(9):1491-501.

144. Lim CP, Harrison RF, Kennedy RL. Application of autonomous neural network systems to medical pattern classification tasks. Artificial intelligence in medicine. 1997;11(3):215-39.

145. Lin K, Xie JQ, Hu YH, Kong GL. Application of support vector machine in predicting in-hospital mortality risk of patients with acute kidney injury in ICU. Beijing da xue xue bao Yi xue ban = Journal of Peking University Health sciences. 2018;50(2):239-44.

146. Lin R, Stanley MD, Ghassemi MM, Nemati S. A Deep Deterministic Policy Gradient Approach to Medication Dosing and Surveillance in the ICU. Conference proceedings : Annual International Conference of the IEEE Engineering in Medicine and Biology Society IEEE Engineering in Medicine and Biology Society Annual Conference. 2018;2018:4927-31.

147. Lin SP, Lee CH, Lu YS, Hsu LN. A comparison of MICU survival prediction using the logistic regression model and artificial neural network model. J Nurs Res. 2006;14(4):306-14.

148. Liu C-L, Soong R-S, Lee W-C, Chen D-H, Hsu S-H. A predictive model for acute allograft rejection of liver transplantation. Expert Systems with Applications. 2018;94:228-36.

149. Liu J, Chen XX, Fang L, Li JX, Yang T, Zhan Q, et al. Mortality prediction based on imbalanced high-dimensional ICU big data. Computers in Industry. 2018;98:218-25.

150. Liu Y, Traskin M, Lorch SA, George EI, Small D. Ensemble of trees approaches to risk adjustment for evaluating a hospital's performance. Health Care Management Science. 2015;18(1):58-66.

151. Luaces O, Taboada F, Albaiceta GM, Dominguez LA, Enriquez P, Bahamonde A, et al. Predicting the probability of survival in intensive care unit patients from a small number of variables and training examples. Artificial Intelligence in Medicine. 2009;45(1):63-76.

152. Lukaszewski RA, Yates AM, Jackson MC, Swingler K, Scherer JM, Simpson AJ, et al. Presymptomatic prediction of sepsis in intensive care unit patients. Clin Vaccine Immunol. 2008;15(7):1089-94.

153. M AFP, Santos MD, Springer DB, Clifford GD. Heart beat detection in multimodal physiological data using a hidden semi-Markov model and signal quality indices. Physiological Measurement. 2015;36(8):1717-27.

154. Maglaveras N, Stamkopoulos T, Pappas C, Strintzis MG. An adaptive backpropagation neural network for real-time ischemia episodes detection: development and performance analysis using the European ST-T database. IEEE Transactions on Biomedical Engineering. 1998;45(7):805-13.

155. Maharlou H, Kalhori SRN, Shahbazi S, Ravangard R. Predicting Length of Stay in Intensive Care Units after Cardiac Surgery: Comparison of Artificial Neural Networks and Adaptive Neuro-fuzzy System. Healthcare Informatics Research. 2018;24(2):109-17.

156. Mani S, Ozdas A, Aliferis C, Varol HA, Chen Q, Carnevale R, et al. Medical decision support using machine learning for early detection of late-onset neonatal sepsis. Journal of the American Medical Informatics Association. 2014;21(2):326-36.

157. Mao Q, Jay M, Hoffman JL, Calvert J, Barton C, Shimabukuro D, et al. Multicentre validation of a sepsis prediction algorithm using only vital sign data in the emergency department, general ward and ICU. BMJ Open. 2018;8(1):e017833.

158. Marafino BJ, Boscardin WJ, Dudley RA. Efficient and sparse feature selection for biomedical text classification via the elastic net: Application to ICU risk stratification from nursing notes. Journal of Biomedical Informatics. 2015;54:114-20.

159. Marafino BJ, Davies JM, Bardach NS, Dean ML, Dudley RA. N-gram support vector machines for scalable procedure and diagnosis classification, with applications to clinical free text data from the intensive care unit. Journal of the American Medical Informatics Association. 2014;21(5):871-5.

160. Martin-Gonzalez F, Gonzalez-Robledo J, Sanchez-Hernandez F, Moreno-Garcia MN. Success/Failure Prediction of Noninvasive Mechanical Ventilation in Intensive Care Units. Using Multiclassifiers and Feature Selection Methods. Methods of Information in Medicine. 2016;55(3):234-41.

161. Mayaud L, Lai PS, Clifford GD, Tarassenko L, Celi LA, Annane D. Dynamic Data During Hypotensive Episode Improves Mortality Predictions Among Patients With Sepsis and Hypotension. Critical Care Medicine. 2013;41(4):954-62.

162. McGeachie MJ, Sordillo JE, Gibson T, Weinstock GM, Liu YY, Gold DR, et al. Longitudinal Prediction of the Infant Gut Microbiome with Dynamic Bayesian Networks. Scientific Reports. 2016;6:20359.

163. Megjhani M, Terilli K, Frey H-P, Velazquez AG, Doyle KW, Connolly ES, et al. Incorporating High-Frequency Physiologic Data Using Computational Dictionary Learning Improves Prediction of Delayed Cerebral Ischemia Compared to Existing Methods. Frontiers in Neurology. 2018;9.

164. Meiring C, Dixit A, Harris S, MacCallum NS, Brealey DA, Watkinson PJ, et al. Optimal intensive care outcome prediction over time using machine learning. Plos One. 2018;13(11).

165. Mesin L, Costa P. Prognostic value of EEG indexes for the Glasgow outcome scale of comatose patients in the acute phase. J Clin Monit Comput. 2014;28(4):377-85.

166. Meyer A, Zverinski D, Pfahringer B, Kempfert J, Kuehne T, Sundermann SH, et al. Machine learning for real-time prediction of complications in critical care: a retrospective study. Lancet Respiratory Medicine. 2018;6(12):905-14.

167. Meyfroidt G, Guiza F, Cottem D, De Becker W, Van Loon K, Aerts JM, et al. Computerized prediction of intensive care unit discharge after cardiac surgery: development and validation of a Gaussian processes model. BMC Med Inf Decis Mak. 2011;11:64.

168. Michel E, Zernikow B. Can PRISM predict length of PICU stay? An analysis of 2000 cases. Medical Informatics and the Internet in Medicine. 2003;28(3):209-19.

169. Mikhno A, Ennett CM. Prediction of extubation failure for neonates with respiratory distress syndrome using the MIMIC-II clinical database. Conf Proc IEEE Eng Med Biol Soc. 2012;2012:5094-7.

170. Mobley BA, Leasure R, Davidson L. Artificial neural network predictions of lengths of stay on a post-coronary care unit. Heart & Lung. 1995;24(3):251-6.

171. Mohamadlou H, Lynn-Palevsky A, Barton C, Chettipally U, Shieh L, Calvert J, et al. Prediction of Acute Kidney Injury With a Machine Learning Algorithm Using Electronic Health Record Data. Can. 2018;5:2054358118776326.

172. Monasterio V, Burgess F, Clifford GD. Robust classification of neonatal apnoea-related desaturations. Physiological Measurement. 2012;33(9):1503-16.

173. Moridani MK, Setarehdan SK, Nasrabadi AM, Hajinasrollah E. New algorithm of mortality risk prediction for cardiovascular patients admitted in intensive care unit. International journal of clinical and experimental medicine. 2015;8(6):8916-26.

174. Morik K, Imhoff M, Brockhausen P, Joachims T, Gather U. Knowledge discovery and knowledge validation in intensive care.[Erratum appears in Artif Intell Med 2000 Oct;20(2):following 178 Note: Imboff, M [corrected to Imhoff, M]]. Artificial Intelligence in Medicine. 2000;19(3):225-49.

175. Mueller M, Wagner CL, Annibale DJ, Knapp RG, Hulsey TC, Almeida JS. Parameter selection for and implementation of a web-based decision-support tool to predict extubation outcome in premature infants. BMC Med Inf Decis Mak. 2006;6:11.

176. Nagaraj SB, Biswal S, Boyle EJ, Zhou DW, McClain LM, Bajwa EK, et al. Patient-Specific Classification of ICU Sedation Levels From Heart Rate Variability. Critical Care Medicine. 2017;45(7):e683-e90.

177. Nagaraj SB, McClain LM, Boyle EJ, Zhou DW, Ramaswamy SM, Biswal S, et al. Electroencephalogram Based Detection of Deep Sedation in ICU Patients Using Atomic Decomposition. Ieee Transactions on Biomedical Engineering. 2018;65(12):2684-91.

178. Nagaraj SB, McClain LM, Zhou DW, Biswal S, Rosenthal ES, Purdon PL, et al. Automatic Classification of Sedation Levels in ICU Patients Using Heart Rate Variability. Critical Care Medicine. 2016;44(9):e782-9.

179. Nagaraj SB, Ramaswamy SM, Biswal S, Boyle EJ, Zhou DW, McClain LM, et al. Heart rate variability as a biomarker for sedation depth estimation in ICU patients. Conf Proc IEEE Eng Med Biol Soc. 2016;2016:6397-400.

180. Nanayakkara S, Fogarty S, Tremeer M, Ross K, Richards B, Bergmeir C, et al. Characterising risk of in-hospital mortality following cardiac arrest using machine learning: A retrospective international registry study. PLoS medicine. 2018;15(11):e1002709-e.

181. Nelson DW, Bellander BM, Maccallum RM, Axelsson J, Alm M, Wallin M, et al. Cerebral microdialysis of patients with severe traumatic brain injury exhibits highly individualistic patterns as visualized by cluster analysis with self-organizing maps. Critical Care Medicine. 2004;32(12):2428-36.

182. Nemati S, Holder A, Razmi F, Stanley MD, Clifford GD, Buchman TG. An Interpretable Machine Learning Model for Accurate Prediction of Sepsis in the ICU. Critical Care Medicine. 2017;26:26.

183. Ngufor C, Murphree D, Upadhyaya S, Madde N, Pathak J, Carter R, et al. Predicting Prolonged Stay in the ICU Attributable to Bleeding in Patients Offered Plasma Transfusion. AMIA Annual Symposium proceedings AMIA Symposium. 2016;2016:954-63.

184. Nikiforidis GC, Sakellaropoulos GC. Expert system support using Bayesian belief networks in the prognosis of head-injured patients of the ICU. Medical Informatics. 1998;23(1):1-18.

185. Oh J, Cho D, Park J, Na SH, Kim J, Heo J, et al. Prediction and early detection of delirium in the intensive care unit by using heart rate variability and machine learning. Physiological measurement. 2018.

186. Olive MK, Owens GE. Current monitoring and innovative predictive modeling to improve care in the pediatric cardiac intensive care unit. Translational Pediatrics. 2018;7(2):120-8.

187. Ongenae F, Van Looy S, Verstraeten D, Verplancke T, Benoit D, De Turck F, et al. Time series classification for the prediction of dialysis in critically ill patients using echo state networks. Engineering Applications of Artificial Intelligence. 2013;26(3):984-96.

188. Otero A, Felix P, Barro S, Palacios F. Addressing the flaws of current critical alarms: a fuzzy constraint satisfaction approach. Artificial Intelligence in Medicine. 2009;47(3):219-38.

189. Paetz J. Knowledge-based approach to septic shock patient data using a neural network with trapezoidal activation functions. Artificial Intelligence in Medicine. 2003;28(2):207-30.

190. Papadelis C, Ashkezari SF, Doshi C, Thome-Souza S, Pearl PL, Grant PE, et al. Real-time multi-channel monitoring of burst-suppression using neural network technology during pediatric status epilepticus treatment. Clin Neurophysiol. 2016;127(8):2820-31.

191. Pappada SM, Borst MJ, Cameron BD, Bourey RE, Lather JD, Shipp D, et al. Development of a neural network model for predicting glucose levels in a surgical critical care setting. Patient Saf Surg. 2010;4(1):15.

192. Pappada SM, Cameron BD, Tulman DB, Bourey RE, Borst MJ, Olorunto W, et al. Evaluation of a model for glycemic prediction in critically ill surgical patients. PLoS ONE [Electronic Resource]. 2013;8(7):e69475.

193. Paradkar N, Chowdhury SR. Coronary artery disease detection using photoplethysmography. Conference proceedings : Annual International Conference of the IEEE Engineering in Medicine and Biology Society IEEE Engineering in Medicine and Biology Society Annual Conference. 2017;2017:100-3.

194. Park S, Megjhani M, Frey H-P, Grave E, Wiggins C, Terilli KL, et al. Predicting delayed cerebral ischemia after subarachnoid hemorrhage using physiological time series data. Journal of clinical monitoring and computing. 2018.

195. Parreco J, Hidalgo A, Parks JJ, Kozol R, Rattan R. Using artificial intelligence to predict prolonged mechanical ventilation and tracheostomy placement. J Surg Res. 2018;228:179-87.

196. Parreco JP, Hidalgo AE, Badilla AD, Ilyas O, Rattan R. Predicting central line-associated bloodstream infections and mortality using supervised machine learning. J Crit Care. 2018;45:156-62.

197. Peixoto R, Ribeiro L, Portela F, Santos MF, Rua F. "Predicting Resurgery in Intensive Care - A data Mining Approach". In: Shakshuki E, editor. 8th International Conference on Emerging Ubiquitous Systems and Pervasive Networks. Procedia Computer Science. 1132017. p. 577-84.

198. Pereira RD, Salgado CM, Dejam A, Reti SR, Vieira SM, Sousa JM, et al. Fuzzy Modeling to Predict Severely Depressed Left Ventricular Ejection Fraction following Admission to the Intensive Care Unit Using Clinical Physiology. ScientificWorldJournal. 2015;2015:212703.

199. Pirracchio R, Petersen ML, Carone M, Rigon MR, Chevret S, van der Laan MJ. Mortality prediction in intensive care units with the Super ICU Learner Algorithm (SICULA): a population-based study. Lancet Respir Med. 2015;3(1):42-52.

200. Pomi A, Olivera F. Context-sensitive autoassociative memories as expert systems in medical diagnosis. BMC Med Inf Decis Mak. 2006;6:39.

201. Precup D, Robles-Rubio CA, Brown KA, Kanbar L, Kaczmarek J, Chawla S, et al. Prediction of extubation readiness in extreme preterm infants based on measures of cardiorespiratory variability. Conf Proc IEEE Eng Med Biol Soc. 2012;2012:5630-3.

202. Rehm GB, Hang J, Kuhn BT, Delplanque J-P, Anderson NR, Adams JY, et al. Creation of a Robust and Generalizable Machine Learning Classifier for Patient Ventilator Asynchrony. Methods of Information in Medicine. 2018;57(4):208-19.

203. Revuelta-Zamorano P, Sanchez A, Luis Rojo-Alvarez J, Alvarez-Rodriguez J, Ramos-Lopez J, Soguero-Ruiz C. Prediction of Healthcare Associated Infections in an Intensive Care Unit Using Machine Learning and Big Data Tools. In: Kyriacou E, Christofides S, Pattichis CS, editors. Xiv Mediterranean Conference on Medical and Biological Engineering and Computing 2016. IFMBE Proceedings. 572016. p. 834-9.

204. Reyes-Garcia J, Galeana-Zapien H, Galaviz-Mosqueda A, Torres-Huitzil C. Evaluation of the Impact of Data Uncertainty on the Prediction of Physiological Patient Deterioration. Ieee Access. 2018;6:38595-606.

205. Ribas Ripoll VJ, Vellido A, Romero E, Ruiz-Rodriguez JC. Sepsis mortality prediction with the Quotient Basis Kernel. Artificial Intelligence in Medicine. 2014;61(1):45-52.

206. Rocha T, Paredes S, Carvalho P, Henriques J, Harris M. Wavelet based time series forecast with application to acute hypotensive episodes prediction. Conf Proc IEEE Eng Med Biol Soc. 2010;2010:2403-6.

207. Rocha T, Paredes S, de Carvalho P, Henriques J. Prediction of acute hypotensive episodes by means of neural network multi-models. Comput Biol Med. 2011;41(10):881-90.

208. Roumani YF, May JH, Strum DP, Vargas LG. Classifying highly imbalanced ICU data. Health Care Management Science. 2013;16(2):119-28.

209. Rowan M, Ryan T, Hegarty F, O'Hare N. The use of artificial neural networks to stratify the length of stay of cardiac patients based on preoperative and initial postoperative factors. Artificial Intelligence in Medicine. 2007;40(3):211-21.

210. Ruyssinck J, van der Herten J, Houthooft R, Ongenae F, Couckuyt I, Gadeyne B, et al. Random Survival Forests for Predicting the Bed Occupancy in the Intensive Care Unit. Comput. 2016;2016:7087053.

211. Saadah LM, Chedid FD, Sohail MR, Nazzal YM, Al Kaabi MR, Rahmani AY. Palivizumab prophylaxis during nosocomial outbreaks of respiratory syncytial virus in a neonatal intensive care unit: predicting effectiveness with an artificial neural network model. Pharmacotherapy. 2014;34(3):251-9.

212. Salgado CM, Ferreira MC, Vieira SM. Mixed Fuzzy Clustering for Misaligned Time Series. Ieee Transactions on Fuzzy Systems. 2017;25(6):1777-94.

213. Samanta B, Bird GL, Kuijpers M, Zimmerman RA, Jarvik GP, Wernovsky G, et al. Prediction of periventricular leukomalacia. Part I: Selection of hemodynamic features using logistic regression and decision tree algorithms. Artificial Intelligence in Medicine. 2009;46(3):201-15.

214. Saqib M, Sha Y, Wang MD. Early Prediction of Sepsis in EMR Records Using Traditional ML Techniques and Deep Learning LSTM Networks. Conf Proc IEEE Eng Med Biol Soc. 2018;2018:4038-41.

215. Saria S, Rajani AK, Gould J, Koller D, Penn AA. Integration of early physiological responses predicts later illness severity in preterm infants. Sci Transl Med. 2010;2(48):48ra65.

216. Savin I, Ershova K, Kurdyumova N, Ershova O, Khomenko O, Danilov G, et al. Healthcare-associated ventriculitis and meningitis in a neuro-ICU: Incidence and risk factors selected by machine learning approach. J Crit Care. 2018;45:95-104.

217. Scalzo F, Hu X. Semi-supervised detection of intracranial pressure alarms using waveform dynamics. Physiological Measurement. 2013;34(4):465-78.

218. Scalzo F, Liebeskind D, Hu X. Reducing false intracranial pressure alarms using morphological waveform features. IEEE Transactions on Biomedical Engineering. 2013;60(1):235-9.

219. Scicluna BP, van Vught LA, Zwinderman AH, Wiewel MA, Davenport EE, Burnham KL, et al. Classification of patients with sepsis according to blood genomic endotype: a prospective cohort study. Lancet Respir Med. 2017;5(10):816-26.

220. Sharafoddini A, Dubin JA, Maslove DM, Lee J. A New Insight Into Missing Data in Intensive Care Unit Patient Profiles: Observational Study. JMIR Medical Informatics. 2019;7(1):e11605.

221. Shashikumar SP, Stanley MD, Sadiq I, Li Q, Holder A, Clifford GD, et al. Early sepsis detection in critical care patients using multiscale blood pressure and heart rate dynamics. Journal of Electrocardiology. 2017;50(6):739-43.

222. Shimabukuro DW, Barton CW, Feldman MD, Mataraso SJ, Das R. Effect of a machine learning-based severe sepsis prediction algorithm on patient survival and hospital length of stay: a randomised clinical trial. BMJ Open Respir Res. 2017;4(1):e000234.

223. Silva A, Cortez P, Santos MF, Gomes L, Neves J. Mortality assessment in intensive care units via adverse events using artificial neural networks. Artificial Intelligence in Medicine. 2006;36(3):223-34.

224. Silva A, Cortez P, Santos MF, Gomes L, Neves J. Rating organ failure via adverse events using data mining in the intensive care unit. Artificial Intelligence in Medicine. 2008;43(3):179-93.

225. Sottile PD, Albers D, Higgins C, McKeehan J, Moss MM. The Association Between Ventilator Dyssynchrony, Delivered Tidal Volume, and Sedation Using a Novel Automated Ventilator Dyssynchrony Detection Algorithm. Critical Care Medicine. 2018;46(2):e151-e7.

226. Spencer RG, Lessard CS, Davila F, Etter B. Self-organising discovery, recognition and prediction of haemodynamic patterns in the intensive care unit. Medical & Biological Engineering & Computing. 1997;35(2):117-23.

227. Sriraam N. EEG based automated detection of auditory loss: A pilot study. Expert Systems with Applications. 2012;39(1):723-31.

228. Stein DM, Hu PF, Chen HH, Yang S, Stansbury LG, Scalea TM. Computational gene mapping to analyze continuous automated physiologic monitoring data in neuro-trauma intensive care. Journal of Trauma and Acute Care Surgery. 2012;73(2):419-24.

229. Suka M, Oeda S, Ichimura T, Yoshida K, Takezawa J. Comparison of proportional hazard model and neural network models in a real data set of intensive care unit patients. Stud Health Technol Inform. 2004;107(Pt 1):741-5.

230. Sun H, Nagaraj SB, Akeju O, Purdon PL, Westover BM. Brain Monitoring of Sedation in the Intensive Care Unit Using a Recurrent Neural Network. Conference proceedings : Annual International Conference of the IEEE Engineering in Medicine and Biology Society IEEE Engineering in Medicine and Biology Society Annual Conference. 2018;2018:1-4.

231. Swiercz M, Mariak Z, Lewko J, Chojnacki K, Kozlowski A, Piekarski P. Neural network technique for detecting emergency states in neurosurgical patients. Medical & Biological Engineering & Computing. 1998;36(6):717-22.

232. Tang BM, McLean AS, Dawes IW, Huang SJ, Lin RC. Gene-expression profiling of peripheral blood mononuclear cells in sepsis. Critical Care Medicine. 2009;37(3):882-8.

233. Temko A, Thomas E, Marnane W, Lightbody G, Boylan G. EEG-based neonatal seizure detection with Support Vector Machines. Clin Neurophysiol. 2011;122(3):464-73.

234. Temko A, Thomas E, Marnane W, Lightbody G, Boylan GB. Performance assessment for EEG-based neonatal seizure detectors. Clin Neurophysiol. 2011;122(3):474-82.

235. Tjepkema-Cloostermans MC, Hofmeijer J, Beishuizen A, Hom HW, Blans MJ, Bosch FH, et al. Cerebral Recovery Index: Reliable Help for Prediction of Neurologic Outcome After Cardiac Arrest. Critical Care Medicine. 2017;45(8):e789-e97.

236. Toltzis P, Soto-Campos G, Kuhn EM, Hahn R, Kanter RK, Wetzel RC. Evidence-Based Pediatric Outcome Predictors to Guide the Allocation of Critical Care Resources in a Mass Casualty Event. Pediatric Critical Care Medicine. 2015;16(7):E207-E16.

237. Tong Y, Frize M, Walker R. Extending ventilation duration estimations approach from adult to neonatal intensive care patients using artificial neural networks. IEEE Trans Inf Technol Biomed. 2002;6(2):188-91.

238. Tu JV, Guerriere MR. Use of a neural network as a predictive instrument for length of stay in the intensive care unit following cardiac surgery. Comput Biomed Res. 1993;26(3):220-9.

239. Van Loon K, Guiza F, Meyfroidt G, Aerts JM, Ramon J, Blockeel H, et al. Dynamic data analysis and data mining for prediction of clinical stability. Stud Health Technol Inform. 2009;150:590-4.

240. Van Looy S, Verplancke T, Benoit D, Hoste E, Van Maele G, De Turck F, et al. A novel approach for prediction of tacrolimus blood concentration in liver transplantation patients in the intensive care unit through support vector regression. Crit Care. 2007;11(4):R83.

241. Van Poucke S, Zhang Z, Schmitz M, Vukicevic M, Laenen MV, Celi LA, et al. Scalable Predictive Analysis in Critically Ill Patients Using a Visual Open Data Analysis Platform. PLoS ONE [Electronic Resource]. 2016;11(1):e0145791.

242. Van Steenkiste T, Ruyssinck J, De Baets L, Decruyenaere J, De Turck F, Ongenae F, et al. Accurate prediction of blood culture outcome in the intensive care unit using long short-term memory neural networks. Artificial intelligence in medicine. 2018.

243. Vanhoutte KJ, Laarakkers C, Marchiori E, Pickkers P, Wetzels JF, Willems JL, et al. Biomarker discovery with SELDI-TOF MS in human urine associated with early renal injury: evaluation with computational analytical tools. Nephrol Dial Transplant. 2007;22(10):2932-43.

244. Velasevic DM, Saletic DZ, Saletic SZ. A fuzzy sets theory application in determining the severity of respiratory failure. International Journal of Medical Informatics. 2001;63(1-2):101-7.

245. Vellido A, Ribas V, Morales C, Ruiz Sanmartin A, Ruiz Rodriguez JC. Machine learning in critical care: state-of-the-art and a sepsis case study. Biomedical Engineering Online. 2018;17.

246. Venugopalan J, Chanani N, Maher K, Wang MD. Combination of static and temporal data analysis to predict mortality and readmission in the intensive care. Conf Proc IEEE Eng Med Biol Soc. 2017;2017:2570-3.

247. Verive MJ, Irazuzta J, Steinhart CM, Orlowski JP, Jaimovich DG. Evaluating the frequency rate of hypomagnesemia in critically ill pediatric patients by using multiple regression analysis and a computer-based neural network. Critical Care Medicine. 2000;28(10):3534-9.

248. Verplancke T, Van Looy S, Benoit D, Vansteelandt S, Depuydt P, De Turck F, et al. Support vector machine versus logistic regression modeling for prediction of hospital mortality in critically ill patients with haematological malignancies. BMC Med Inf Decis Mak. 2008;8:56.

249. Verplancke T, Van Looy S, Steurbaut K, Benoit D, De Turck F, De Moor G, et al. A novel time series analysis approach for prediction of dialysis in critically ill patients using echo-state networks. BMC Med Inf Decis Mak. 2010;10:4.

250. Veselis RA, Reinsel R, Sommer S, Carlon G. USE OF NEURAL NETWORK ANALYSIS TO CLASSIFY ELECTROENCEPHALOGRAPHIC PATTERNS AGAINST DEPTH OF MIDAZOLAM SEDATION IN INTENSIVE-CARE UNIT PATIENTS. Journal of Clinical Monitoring. 1991;7(3):259-67.

251. Vranas KC, Jopling JK, Sweeney TE, Ramsey MC, Milstein AS, Slatore CG, et al. Identifying Distinct Subgroups of ICU Patients: A Machine Learning Approach. Critical Care Medicine. 2017;45(10):1607-15.

252. Walczak S, Scorpio RJ. Predicting pediatric length of stay and acuity of care in the first ten minutes with artificial neural networks. Pediatric Critical Care Medicine. 2000;1(1):42-7.

253. Wong LSS, Young JD. A comparison of ICU mortality prediction using the APACHE II scoring system and artificial neural networks. Anaesthesia. 1999;54(11):1048-54.

254. Wong RS, Ismail NA. An Application of Bayesian Approach in Modeling Risk of Death in an Intensive Care Unit. PLoS ONE [Electronic Resource]. 2016;11(3):e0151949.

255. Xiao R, King J, Villaroman A, Do DH, Boyle NG, Hu X. Predict In-Hospital Code Blue Events using Monitor Alarms through Deep Learning Approach. Conf Proc IEEE Eng Med Biol Soc. 2018;2018:3717-20.

256. Xu H, Wu W, Nemati S, Zha H. Patient Flow Prediction via Discriminative Learning of Mutually-Correcting Processes. Ieee Transactions on Knowledge and Data Engineering. 2017;29(1):157-71.

257. Yamamura S, Takehira R, Kawada K, Nishizawa K, Katayama S, Hirano M, et al. Application of artificial neural network modelling to identify severely ill patients whose aminoglycoside concentrations are likely to fall below therapeutic concentrations. J Clin Pharm Ther. 2003;28(5):425-32.

258. Zhang Y, Szolovits P. Patient-specific learning in real time for adaptive monitoring in critical care. Journal of Biomedical Informatics. 2008;41(3):452-60.
